# Facilitation of AMPA Receptor Synaptic Delivery as a Molecular Mechanism for Cognitive Enhancement

**DOI:** 10.1371/journal.pbio.1001262

**Published:** 2012-02-21

**Authors:** Shira Knafo, César Venero, Cristina Sánchez-Puelles, Inmaculada Pereda-Peréz, Ana Franco, Carmen Sandi, Luz M. Suárez, José M. Solís, Lidia Alonso-Nanclares, Eduardo D. Martín, Paula Merino-Serrais, Erika Borcel, Shizhong Li, Yongshuo Chen, Juncal Gonzalez-Soriano, Vladimir Berezin, Elisabeth Bock, Javier DeFelipe, José A. Esteban

**Affiliations:** 1 Centro de Biología Molecular “Severo Ochoa,” Consejo Superior de Investigaciones Científicas (CSIC)/Universidad Autónoma de Madrid, Madrid, Spain; 2 Instituto Cajal (CSIC), Centro de Tecnología Biomédica, Universidad Politécnica de Madrid, and Centro de Investigación Biomédica en Red sobre Enfermedades Neurodegenerativas (CIBERNED), Madrid, Spain; 3 Department of Psychobiology, Universidad Nacional de Educación a Distancia, Madrid, Spain; 4 Centro Nacional Biotecnología (CSIC), Universidad Autónoma de Madrid, Madrid, Spain; 5 Brain Mind Institute, Ecole Polytechnique Federale de Lausanne (EPFL), Switzerland; 6 Servicio de Neurobiología-Investigación, Hospital Ramón y Cajal, IRYCIS, Madrid, Spain; 7 Departamento de Ciencias Médicas, Universidad de Castilla-la Mancha, Albacete, Spain; 8 Protein Laboratory, Department of Neuroscience and Pharmacology, Faculty of Health Sciences, University of Copenhagen, Copenhagen, Denmark; 9 Department of Anatomy, Faculty of Veterinary Medicine, Complutense University, Madrid, Spain; University of Basel, Switzerland

## Abstract

Cell adhesion molecules and downstream growth factor-dependent signaling are critical for brain development and synaptic plasticity, and they have been linked to cognitive function in adult animals. We have previously developed a mimetic peptide (FGL) from the neural cell adhesion molecule (NCAM) that enhances spatial learning and memory in rats. We have now investigated the cellular and molecular basis of this cognitive enhancement, using biochemical, morphological, electrophysiological, and behavioral analyses. We have found that FGL triggers a long-lasting enhancement of synaptic transmission in hippocampal CA1 neurons. This effect is mediated by a facilitated synaptic delivery of AMPA receptors, which is accompanied by enhanced NMDA receptor-dependent long-term potentiation (LTP). Both LTP and cognitive enhancement are mediated by an initial PKC activation, which is followed by persistent CaMKII activation. These results provide a mechanistic link between facilitation of AMPA receptor synaptic delivery and improved hippocampal-dependent learning, induced by a pharmacological cognitive enhancer.

## Abstract 2

A small peptide from a neuronal cell adhesion molecule enhances synaptic plasticity in the hippocampus and results in improved cognitive performance in rats.

## Author Summary

The human brain contains trillions of neuronal connections, called synapses, whose pattern of activity controls all our cognitive functions. These synaptic connections are dynamic and constantly changing in their strength and properties, and this process of synaptic plasticity is essential for learning and memory. Alterations in synaptic plasticity mechanisms are thought to be responsible for multiple cognitive deficits, such as autism, Alzheimer's disease, and several forms of mental retardation. In this study, we show that synapses can be made more plastic using a small protein fragment (peptide) derived from a neuronal protein involved in cell-to-cell communication. This peptide (FGL) initiates a cascade of events inside the neuron that results in the facilitation of synaptic plasticity. Specifically, we find that FGL triggers delivery of a specific type of glutamate receptor (AMPA receptors) to synapses in a region of the brain called the hippocampus, which is known to be involved in multiple forms of learning and memory. Importantly, when this peptide was administered to rats, their ability to learn and retain spatial information was enhanced. Therefore, this work demonstrates that cognitive function can be improved pharmacologically in adult animals by enhancing the plasticity of synaptic connections in the brain.

## Introduction

Activity-dependent synaptic changes, generally termed synaptic plasticity, underlie multiple forms of cognitive function, such as learning and memory [1]. Strong interest has accrued in understanding the molecular and cellular mechanisms underlying these changes. Additionally, it is believed that targeted manipulation of these mechanisms may help facilitate or stabilize synaptic plasticity events, with the aim of potentially improving cognitive function under pathological, or even physiological, conditions.

Multiple genetic manipulations in animal models have been shown to produce cognitive enhancement, defined as improved performance in learning and memory behavioral tasks (see, for example, [2]). In the vast majority of cases, cognitive-enhancing mutations are related to signaling mechanisms associated with synaptic plasticity (reviewed in [3]), thus reinforcing the interpretation of synaptic plasticity as a cellular substrate for learning and memory. Nevertheless, the particular mechanism that links changes in synaptic plasticity with enhanced cognitive function is poorly defined. Additionally, for therapeutic purposes, there is great interest in developing pharmacological approaches, rather than genetic manipulations, that effectively modulate synaptic plasticity pathways in a well-defined manner.

**Figure 1:**
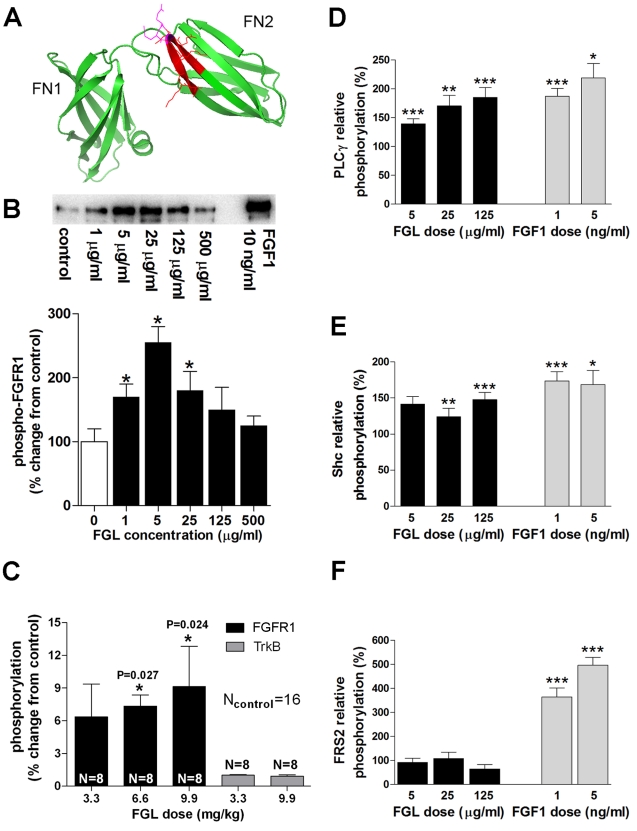
FGL triggers hippocampal FGFR1 phosphorylation in vitro and in vivo. (A) Cartoon structure of the double fibronectin module (FN1+FN2) of human NCAM (Protein Data Bank number 2VKW). The FGL sequence is shown in red with the two glutamine residues critical for the binding to the FGF-receptor highlighted in magenta. (B) Top: Representative immunoblot showing the in vitro phosphorylation of FGFR1 after stimulation of Trex293 cells that express Strep-tagged human FGFR1 with different concentrations of FGL and 10 ng/ml FGF1 (positive control) for 20 min. Bottom: Quantification of FGFR1 phosphorylation by FGL was performed by densitometric analysis of band intensity from four independent experiments similar to the one shown in the upper panel. (C) Phosphorylation of FGFR1 and TrkB was examined from hippocampal homogenates with an enzyme-linked immunosorbent assay (ELISA) 1 h after FGL subcutaneous injection. *N*, number of animals. Results are expressed as percentage ± SEM, with untreated controls set at 0%. (D–F) Phosphorylation of PLCγ (D), Shc (E), and FRS2 (F) in vitro was examined by Western blot, as described in Figure 1B. Treatment with FGF1 served as the positive control. Results from four independent experiments are expressed as a percentage ± SEM, with untreated controls set at 100%. *p<0.05, **p<0.01, ***p<0.001 compared with controls. Statistics were carried out according to the *t* test.

Cell adhesion molecules are well-known effectors of neuronal development and structural plasticity [4]. Some of them have also been linked to synaptic plasticity, learning, and memory [5], particularly via their interaction with growth factor-mediated signaling [6]. From this point of view, cell adhesion molecules are being considered as potential therapeutic targets for the development of pharmacological cognitive enhancers. This is the case of neural cell adhesion molecule (NCAM) [5]. NCAM activity is essential for both early synaptogenesis and synaptic maturation [4], and it influences the strength of excitatory synapses in an activity-dependent manner [7]. NCAM is a member of the immunoglobulin (Ig) superfamily, containing five *N*-terminal Ig modules followed by two fibronectin type III (F3) modules. NCAM is involved in homophilic and heterophilic interactions, as well as in the activation of various signal transduction pathways [8]. Importantly, some of the functions of NCAM in cell remodeling and growth are mediated by fibroblast growth factor receptors (FGFRs). In fact, NCAM interacts with extracellular domains of FGFR to modulate FGFR-dependent intracellular signaling [9]. Based on the functional interplay between NCAM and FGFR, we engineered a synthetic NCAM mimetic peptide, termed FGLoop (FGL), that encompasses the interaction domain of NCAM with FGFR: F and G β-strands and the interconnecting loop of the second F3 module of NCAM (red ribbon in Figure 1A). We previously showed that FGL elicits FGFR-mediated signaling [10] and induces neuritogenesis and survival in neuronal cultures [11]. Most importantly, we found that in vivo administration of FGL enhances spatial and social memory retention in rats [12],[13]. FGL has also been shown to prevent cognitive impairment induced by stress [14],[15] and oligomeric β-amyloid [16], and have antidepressant-like effects in rats [17]. Therefore, FGL appears to act as a bona fide cognitive enhancer, possibly by engaging NCAM-FGFR-related signaling. However, the synaptic mechanisms by which this cognitive enhancement is produced remain unknown.

Some of the signaling pathways recruited by FGFR activation [18] cross-talk with molecular mechanisms associated with synaptic plasticity, particularly long-term potentiation (LTP). LTP is one of the best characterized forms of synaptic plasticity in the hippocampus and is considered to be a cellular correlate for information storage in the brain during learning and memory processes [1]. A major contributor to synaptic potentiation during LTP is the incorporation of new AMPA-type glutamate receptors (AMPARs) into excitatory synapses via activity-dependent trafficking [19]. Multiple signal transduction pathways have been shown to regulate AMPAR incorporation into synapses during LTP, most notably the pathways controlled by Ca^2+^/calmodulin-dependent protein kinase II (CaMKII) [20], mitogen-activated protein kinase (MAPK) [21], protein kinase C (PKC) [22], and phosphoinositide-3-kinase (PI3K) [23],[24]. These last three pathways (MAPK, PKC, and PI3K) are engaged during FGFR-dependent signaling [18], and therefore, they are attractive candidates to mediate FGL effects on cognitive function.

The present study uncovers specific synaptic mechanisms and signaling pathways responsible for the cognitive enhancement induced by the NCAM-FGFR agonist FGL. In particular, we show here that FGL enhances LTP in hippocampal slices, and it does so by facilitating AMPAR delivery at synapses upon activation of NMDA receptors (NMDARs). These effects are specifically mediated by PKC activation. Furthermore, behavioral testing revealed that this PKC-dependent mechanism mediates the enhanced cognition induced by FGL. Therefore, these results delineate the intracellular signaling and molecular mechanisms that lead to enhanced synaptic plasticity and improved learning and memory caused by a pharmacological cognitive enhancer. In this manner, we have established a mechanistic link between facilitation of AMPAR synaptic delivery and enhanced cognition.

## Results

### FGL Activates FGFR-Dependent Signaling in vivo and Acts as a Cognitive Enhancer

To start characterizing the signaling pathways that mediate the cognitive actions of FGL, we tested the ability of FGL to activate FGFR-dependent signaling in vitro and in vivo. We found that FGL dose-dependently induces FGFR1 phosphorylation in Trex293 cells transfected with human FGFR1 (Figure 1B). Moreover, FGL triggers FGFR1 phosphorylation in vivo in the hippocampus after subcutaneous injection (Figure 1C, black columns) (we have previously shown that FGL crosses the blood-brain barrier [13]). As a control, FGL did not induce phosphorylation of TrkB (Figure 1C, gray columns), the receptor of brain-derived neurotrophic factor (BDNF), which is a potent modulator of activity-dependent synaptic plasticity and shares some signaling pathways with FGFR [25]. Downstream from FGFR phosphorylation, we found in transfected Trex293 cells that FGL triggers the phosphorylation of PLCγ (phospholipase C-γ) and Shc (Src homologous and collagen) (Figure 1D and E), but not FRS2 (FGFR substrate 2) (Figure 1F, black columns), in contrast to FGF1 (Figure 1F, gray columns). Thus, FGL activates a subset of the signaling pathways triggered by FGF.

**Figure 2:**
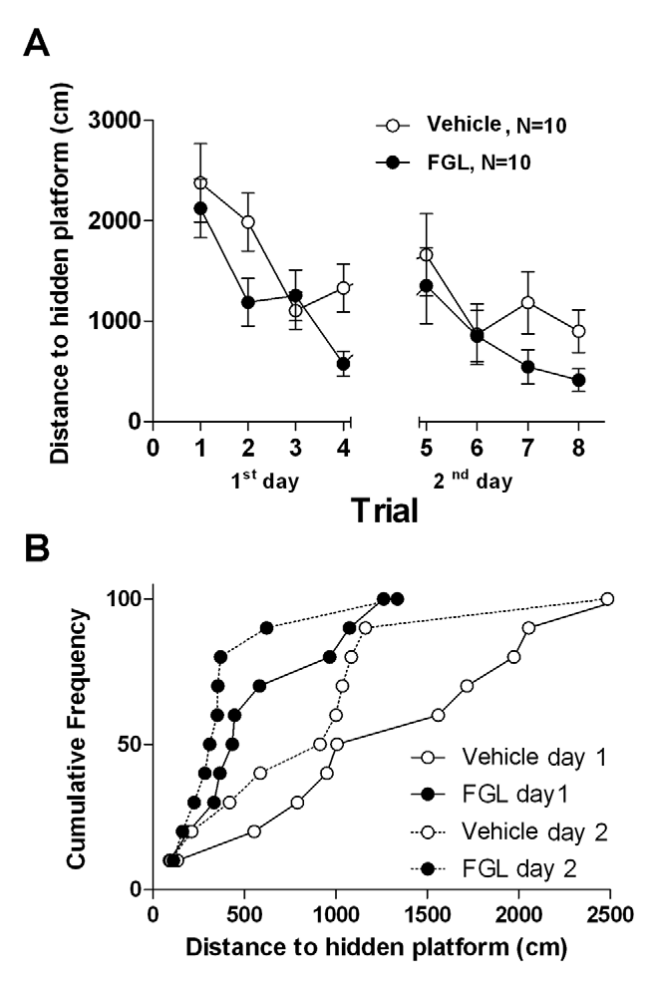
FGL enhances spatial learning. (A) Mean distances swam to find the hidden platform in the Morris water maze are represented for control rats (white symbols) and FGL-treated rats (black symbols) over 2 training days (four trials each). *N*, number of animals. Statistical significance was analyzed with repeated-measures ANOVA. (B) Cumulative frequency distributions of the distances swam by individual rats. Each data point represents the distance swam by one rat in the last trial of each day.

After determining that FGL activates FGFR in vivo and in vitro, we evaluated its cognitive actions. FGL (6.6 mg/kg) or control vehicle (0.9% NaCl) were injected subcutaneously 5 and 2 d before the evaluation of cognitive function. Rats were trained to find a submerged platform in the Morris water maze (see Text S1). Spatial training was performed with the experimenter blind to the treatment of each rat. As shown in Figure 2A, FGL-treated rats outperformed their age-matched controls in this spatial learning task during the 2 d of training (reflected by significantly shorter distances swam to find the hidden platform during the training sessions: *F*_1,18_ = 8.445, *p* = 0.004). This was particularly evident for all tested animals when comparing individual performance during the last trial of each training day (Figure 2B). No significant differences were found in swimming speed between groups (unpublished data), suggesting that FGL does not have peripheral effects.

Therefore, we conclude that FGL triggers FGFR-dependent signaling in the hippocampus in vivo and improves hippocampal-dependent learning when injected peripherally.

### FGL Does Not Alter the Morphology of Dendritic Spines and Synapses

**Figure 3:**
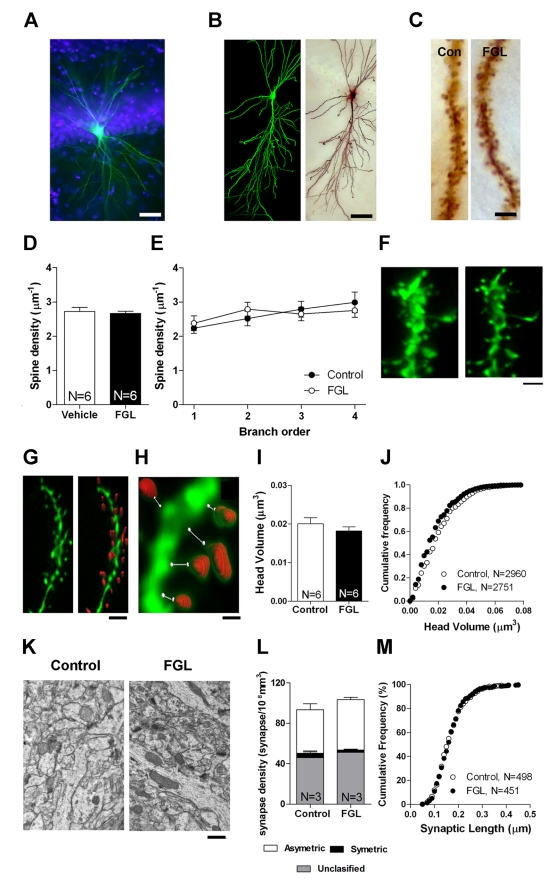
Unchanged morphological parameters after FGL treatment. (A) Fluorescence image of CA1 pyramidal neuron injected with Lucifer Yellow (green). DAPI nuclear staining (blue) was used to facilitate intracellular injection into the soma. Bar = 50 µm. (B) Confocal projection image of CA1 pyramidal neurons (left) and the same neuron processed for DAB staining (right). Bar = 20 µm. (C) High-magnification images of representative spiny dendrites. Bar = 10 µm. (D) Quantification of spine density from stratum radiatum CA1 dendrites. *N*, number of animals. (E) Quantification of spine density sorted by branch order (1–4) of the oblique apical dendrite. (F) Maximum-projection confocal images of a basal CA1 dendritic segment before (left) and after (right) the blind deconvolution protocol (10 iterations). Bar = 1 µm. (G) Maximum-projection image of a dendrite after blind deconvolution (left) and the same dendritic segment with marked spine heads (red) as used to measure head volumes (right). Bar = 2 µm. (H) Higher magnification of a short dendritic segment that shows the measurements of each spine (i.e., spine head volume and neck length). Bar = 0.5 µm. (I) Quantification of spine head volume in FGL and control rats. *N*, number of animals. (J) Cumulative frequency of spine head volume from the same data as in (I). *N*, number of spines. (K) Electron micrographs that show a representative neuropil in the stratum radiatum. Symmetric and asymmetric synapses were identified to quantify synaptic density using unbiased stereology. Bar = 0.5 µm. (L) Quantification of synaptic density in FGL and control rats. *N*, the number of animals. (M) Cumulative frequency of postsynaptic density length. *N*, number of synaptic profiles.

Most glutamatergic excitatory axons establish synapses with dendritic spines of pyramidal neurons, and changes in their density and/or shape are involved in plastic modifications associated with LTP between CA3 and CA1 pyramidal neurons [26]. Additionally, most CA1 pyramidal neurons express FGFR1 [27] and have been strongly implicated in spatial navigation and memory [28]. Thus, as a first step to evaluate the cellular substrate of FGL-induced enhanced cognition, we examined whether dendritic spines in CA1 stratum radiatum are affected by FGL administration. To this end, animals were treated with FGL as described above, and 2 d after the second FGL injection (when cognition enhancement was observed), the animals were perfused with fixative, hippocampal sections were prepared, and CA1 neurons were injected with Lucifer Yellow for analysis of dendritic spine density and morphology (Figure 3A–B). These analyses were performed by experimenters blind to the treatment of the animals. We found no significant differences in spine density (Figure 3C–E), spine head volume (Figure 3F–J), or neck length (Figure S1) between FGL- and vehicle-treated animals. Furthermore, sections adjacent to those used for the intracellular injections were examined at the electron microscope level. As shown in Figure 3K–M, we did not detect changes in synaptic density or cross-sectional lengths of the synaptic junctions, quantified from electron photomicrographs using the size-frequency method [29],[30]. Thus, the cognitive enhancement induced by FGL is not associated with detectable structural changes in CA1 stratum radiatum synapses.

### FGL Enhances Excitatory Synaptic Transmission by Inducing AMPAR Synaptic Delivery

**Figure 4:**
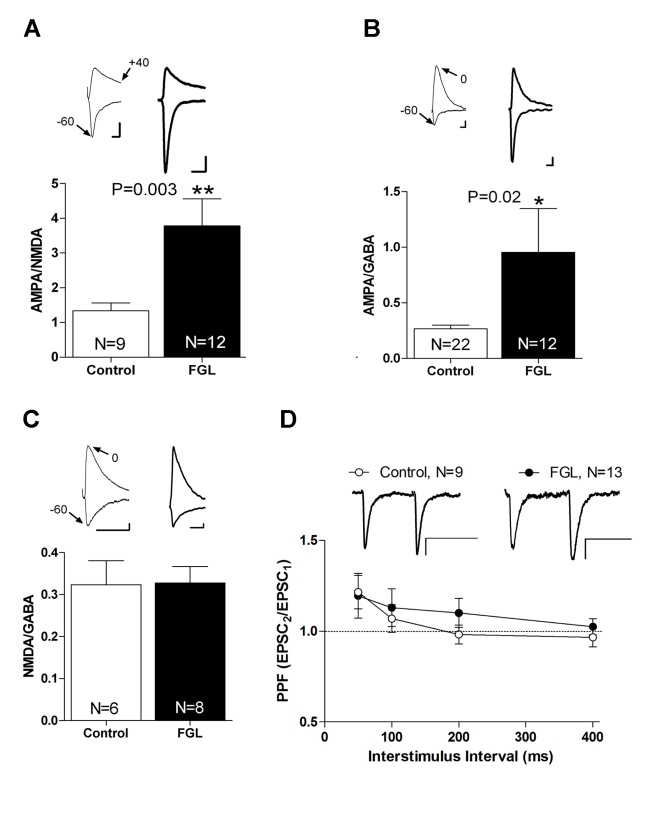
Enhanced postsynaptic excitatory transmission in neurons treated with FGL. (A) Average AMPA/NMDA ratios for treated and untreated cells. AMPAR-mediated responses were recorded at −60 mV, and NMDAR-mediated responses were recorded at +40 mV. The *p* value was determined using the Mann-Whitney test. (B) Average AMPA/GABA ratios for treated and untreated cells. AMPAR-mediated responses were recorded at −60 mV, and GABA-mediated responses were recorded at +0 mV. NMDAR were blocked with DL-AP5. The *p* value was determined using a *t* test. Representative traces appear above the corresponding bars. *N*, number of cells. (C) Average NMDA/GABA ratios for treated and untreated cells. NMDAR-mediated responses were recorded at −60 mV in the absence of Mg^2+^ and in the presence of CNQX to block AMPARs. GABA-mediated responses were recorded at 0 mV. The *p* value was determined using a *t* test. Representative traces appear above the corresponding bars. *N*, number of cells. (D) Paired-pulse facilitation (PPF) in FGL and control neurons. The values denote the ratio of the second EPSC amplitude to the first EPSC amplitude. PPF was tested for 50-, 100-, 200-, and 400-ms interstimulus intervals. Insets. Sample trace of evoked AMPAR-mediated synaptic responses with an interstimulus interval of 50 ms. *N*, number of cells. Scale bars: 10 pA, 50 ms.

We then reasoned that the effect of FGL on cognition may result from functional rather than structural changes in hippocampal synapses. To test the effect of FGL on synaptic transmission, we added FGL (10 µg/ml) to the medium of organotypic hippocampal slice cultures. After 24 h, the culture medium was replaced with fresh medium without FGL, and the slices were kept in culture for an additional 24 h before electrophysiological recordings (see Materials and Methods). Therefore, the electrophysiological responses were evaluated 48 h after the onset of the FGL treatment. This regimen was intended to mimic the in vivo behavioral experiments, in which spatial learning is tested long after FGL has been cleared from cerebrospinal fluid (CSF) [13]. We placed the stimulating electrodes over Schaffer collateral fibers and recorded CA3-to-CA1 synaptic transmission. Synaptic responses were evoked at −60 mV and +40 mV holding potentials, in the presence of the γ-aminobutyric acid-A (GABA_A_) receptor antagonist picrotoxin to obtain separate AMPAR- and NMDAR-mediated responses, and we calculated the ratio between these values (AMPA/NMDA ratio). As shown in Figure 4A, the AMPA/NMDA ratio of synaptic responses significantly increased after FGL treatment compared with control neurons. Similarly, we obtained AMPA/GABA ratios by evoking synaptic responses at −60 mV and 0 mV holding potentials in the presence of the NMDAR antagonist AP5. Similar to AMPA/NMDA ratio, we found a significant increase in the AMPA/GABA ratio after FGL treatment compared with control neurons (Figure 4B). Finally, we calculated NMDA/GABA ratios by recording NMDA responses at −60 mV in the absence of Mg^2+^ and in the presence of CNQX (AMPA receptor antagonist), and GABA responses at 0 mV. As shown in Figure 4C, NMDA/GABA ratios were unaltered by the FGL treatment. Additionally, FGL did not change the presynaptic properties of excitatory transmission assessed by paired-pulse facilitation (Figure 4D), or the passive membrane properties of the neuron, assessed by their input resistance and holding current (Figure S2). In conclusion, these results suggest that FGL produces a functional postsynaptic change at excitatory CA1 synapses, specifically an increase in AMPAR-mediated synaptic transmission.

**Figure 5:**
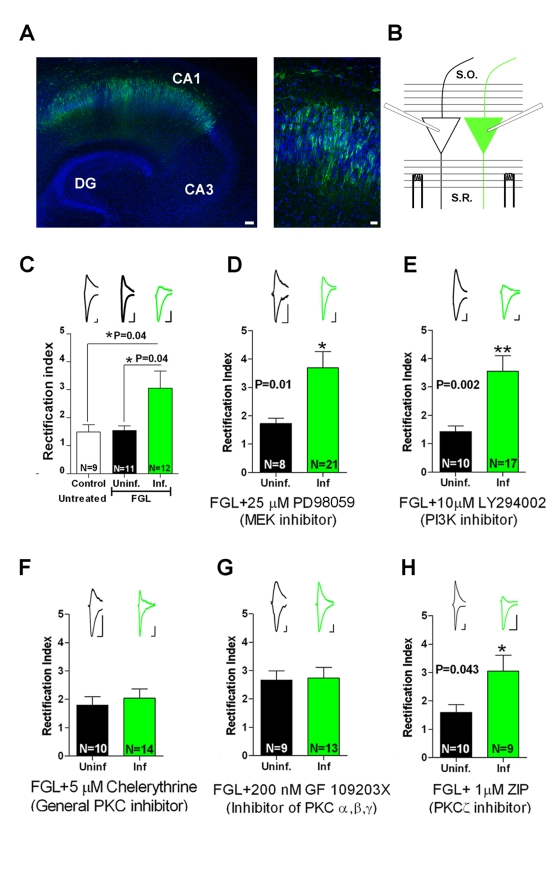
FGL induces AMPA receptor synaptic delivery via PKC activation. (A) Left: CA1 pyramidal neurons that express GluA1-GFP (green) on a DAPI-stained (blue) organotypic slice culture, imaged with laser-scanning confocal microscopy. Bar = 50 µm. Right: High-magnification image of GluA1-GFP-expressing neurons. Bar = 20 µm. (B) Schematic diagram that presents whole-cell recordings obtained from a neuron expressing GluA1-GFP (infected, green) and an adjacent non-fluorescent (uninfected, white) neuron. (C) AMPAR-mediated responses were recorded at −60 mV and +40 mV. The rectification index was calculated as the ratio of responses at these holding potentials. The *p* value was determined using the Mann-Whitney test. (D–H) FGL-induced rectification after incubation with inhibitors of different signal transduction pathways: MEK, PD98059 (D); PI3K, LY294002 (E); PKC, chelerythrine (F); classical PKC isoforms, GF109203X (G); atypical PKC isoforms (H). Sample traces are shown above the corresponding columns of the plot. *N*, number of cells. The *p* value was determined using the Mann-Whitney test. Scale bars = 15 pA and 10 ms.

Enhanced AMPAR synaptic responses may be attributable to an increased number of AMPARs at synapses or a functional modification of preexisting synaptic receptors. To directly determine whether FGL induces the delivery of new AMPARs into synapses, we expressed the GluA1 subunit of AMPARs tagged with GFP (GluAl-GFP) in CA1 neurons in organotypic hippocampal slice cultures (Figure 5A–B). We employed this subunit because it has been previously shown that newly synthesized GluA1-containing AMPARs are not spontaneously inserted at synapses, unless driven by strong synaptic stimulation or activation of specific signaling pathways associated with LTP induction [22],[31]. In addition, overexpressed GluAl-GFP subunits form homomeric AMPARs, whose presence at synapses can be assessed from their inward rectification properties (electrophysiological tagging [31]). GluA1-GFP was expressed in organotypic slice cultures for 60 h (24 h of FGL treatment plus 36 h in fresh medium), and synaptic delivery was quantified as an increase in the ratio of the evoked postsynaptic current at −60 mV relative to the current at +40 mV (rectification index, RI = I_−60_/I_+40_), in the presence of the NMDAR antagonist AP5. We found that FGL treatment increased the rectification index in neurons that express GluA1-GFP (Figure 5C). This result strongly suggests that FGL induces synaptic delivery of AMPARs. To note, FGL treatment alone did not change the rectification index in the absence of GluA1-GFP expression (Figure 5C, “FGL uninfected” versus “Untreated control”), indicating that FGL does not alter the intrinsic rectification properties of endogenous AMPA receptors. Also, GluA1-GFP is not delivered spontaneously into synapses [31].

Altogether, these results indicate that FGL enhances excitatory synaptic transmission by inducing the insertion of new AMPARs at synapses. It is also important to point out that FGL is removed from the culture medium from 24 to 36 h before the electrophysiological recordings, indicating that FGL induces long-lasting changes in synaptic transmission.

### The PKC Pathway Mediates FGL-Induced Synaptic Incorporation of AMPARs

After establishing the specific synaptic modification produced by FGL (enhanced AMPAR synaptic delivery), we identified the underlying signal transduction mechanism. Hippocampal slices were treated with FGL while blocking the three pathways downstream from FGFR activation that may modulate AMPAR trafficking (i.e., the MAPK, PI3K, and PKC pathways). As a reporter for AMPAR synaptic delivery, we monitored inward rectification of synaptic transmission in GluA1-GFP-expressing neurons, described above. Incubation of the slice culture in 25 µM PD98059, a potent inhibitor of MAPK kinase (MEK [32]), did not block the increase in the rectification index induced by FGL in GluA1-GFP-expressing neurons (compare Figure 5C,D). Therefore, we conclude that FGL-induced AMPAR synaptic delivery does not require MAPK activation. Similarly, incubation in 10 µM LY294002, a potent and selective PI3K inhibitor [33], did not prevent GluA1 synaptic delivery (Figure 5E). In contrast, 5 µM chelerythrine, a general inhibitor of PKC [34], did block the FGL-induced increase in rectification (Figure 5F) (the efficacy of chelerythrine as a specific PKC inhibitor is evidenced from the blockade of MARCKS phosphorylation and its translocation from the plasma membrane to the cytosol in response to PKC activation; see Figure S3). This finding indicates that FGL enhances AMPAR synaptic delivery in a PKC-dependent manner. Moreover, incubation of slices with a selective inhibitor of classical PKC isoforms (α, β, and γ) (200 nM GF109203X [35]) also blocked the increase in the rectification index (Figure 5G), whereas a specific inhibitor of atypical PKC isoforms (1 µM zeta inhibitory peptide [ZIP]) did not (Figure 5H).

Therefore, these findings indicated that of the multiple signaling pathways potentially triggered by FGL, the synaptic delivery of AMPARs with the consequent potentiation of synaptic transmission is mediated by PKC activation, specifically by classical PKC isoforms. These results are consistent with the failure of FGL to induce the phosphorylation of FRS2 (Figure 1F), which acts as an important docking platform for the activation of PI3K and MAPK pathways [36],[37].

### FGL Facilitates NMDAR-Dependent LTP in a PKC-Dependent Manner

**Figure 6:**
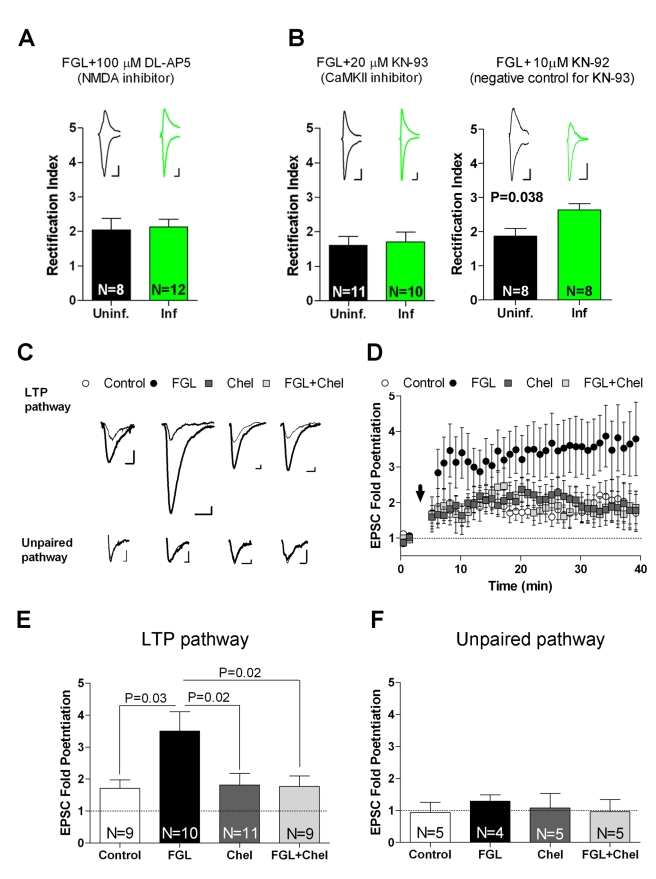
FGL enhances long-term synaptic potentiation. (A–B) Rectification experiments similar to the ones described in Figure 5, after incubation with DL-AP5 (NMDAR inhibitor), KN-93 (CaMKII inhibitor), or KN-92 (inactive analog of KN-93). Sample traces are shown above the graphs. (C) Sample traces of evoked AMPAR-mediated synaptic responses recorded from CA1 neurons at −60 mV before (thin line) and after (thick line) LTP induction. LTP was induced by pairing presynaptic 3 Hz stimulation (540 pulses) with postsynaptic depolarization (0 mV). One of the stimulating electrodes was turned off during LTP induction (“unpaired pathway”). Organotypic slice cultures were incubated with (i) normal culture medium (control), (ii) FGL, (iii) the PKC inhibitor chelerythrine (Chel), or (iv) FGL and chelerythrine (FGL+Chel), as indicated. Treatments were for 24 h and slices were transferred to fresh culture medium (without FGL or chelerythine) for an additional 24 h prior to recordings. (D) Time course of normalized AMPAR-mediated synaptic responses before and after LTP induction (black arrow), from the slices treated as in (C). For simplicity, each time point in the plot corresponds to the average of 12 consecutive stimulations (sampling rate: 0.2 Hz). (E–F) Quantification of average synaptic potentiation from paired (“LTP”) and unpaired pathways from the last 10 min of the time-course shown in (D). The *p* value was determined with the Mann-Whitney test. *N*, number of cells.

The synaptic incorporation of AMPAR induced by FGL may be directly driven by PKC activation and AMPAR phosphorylation [22] or, alternatively, may be an activity-dependent process that is facilitated by FGL. This is an important distinction, because a cognitive enhancer would be expected to modulate synaptic function in a synapse-specific manner. The classic paradigm for activity-dependent synaptic delivery of AMPARs is NMDAR-dependent LTP. Therefore, we hypothesized that FGL induces an LTP-like process in response to spontaneous synaptic activity in hippocampal slices. Notable, spontaneous activity in organotypic slice cultures is typically not sufficient to induce the synaptic delivery of AMPARs [31]. To determine whether FGL-induced synaptic potentiation resembles conventional LTP processes, AMPAR delivery was examined by monitoring inward rectification (described for Figure 5) after blocking NMDARs or CaMKII. We found that 100 µM AP5, a competitive NMDAR antagonist, completely blocked the FGL-induced delivery of tagged AMPARs into synapses (Figure 6A). Similarly, 20 µM KN-93, a potent inhibitor of CaMKII catalytic activity [38], blocked FGL-induced AMPAR synaptic delivery (Figure 6B, left panel). In contrast, the inactive analog KN-92 did not block AMPAR synaptic delivery, as detected by the increase in the rectification index in FGL-treated slices (Figure 6B, right panel). Therefore, FGL-triggered AMPAR delivery depends on the NMDAR and CaMKII activity.

Since FGL-induced synaptic potentiation appears to mimic classic NMDAR-dependent LTP, we decided to test whether FGL affects this form of synaptic plasticity. Similar to the previous experiments, slices were pretreated with FGL for 24 h. The culture medium was then changed (without FGL), and recordings were performed 24 h later. Therefore, as in the previous experiments, electrophysiological experiments start 48 h after the onset of FGL treatment. LTP was induced in CA1 neurons by pairing 3 Hz presynaptic stimulation of the Schaffer collaterals with 0 mV postsynaptic depolarization as previously described [31]. As shown in Figure 6C–E, LTP induction significantly increased AMPAR-mediated responses in both FGL-treated and untreated neurons. Nevertheless, FGL treatment dramatically enhanced LTP expression (3.5-fold potentiation with FGL versus 2-fold potentiation in control neurons; see also Figure S4 for a complete distribution of individual LTP experiments).

To determine whether FGL-induced LTP enhancement occurs through mechanisms similar as FGL-induced GluA1 synaptic delivery, we tested the role of PKC activation in this process. Slices were incubated with 5 µM chelerythrine together with FGL. Electrophysiological recordings were then performed without chelerythrine because PKC activity is required for LTP induction [39]. Notably, the magnitude of LTP after treatment with FGL in the presence of chelerythrine (Figure 6C–E, “FGL+Chel”) was indistinguishable from LTP in untreated neurons, suggesting that PKC activity is required for the enhancing effect of FGL. As a control, neurons treated with chelerythrine alone had similar LTP levels as untreated neurons (Figure 6C–E, “Chel”), indicating that prior PKC activity is not required for subsequent LTP induction. Finally, FGL did not have any effect on the non-potentiated (unpaired) pathway (Figure 6F). As mentioned above, FGL (and chelerythrine) were washed out from the slices 24 h before performing the LTP experiments. Therefore, these data indicate that FGL produces a long-lasting, PKC-dependent enhancement of LTP.

After having established the effect of FGL on synaptic potentiation, we wished to test whether FGL affects LTD, another NMDAR-dependent form of synaptic plasticity. Similar to the previous experiments, slices were pretreated with FGL for 24 h. The culture medium was then changed (without FGL), and recordings were performed 24 h later. LTD was induced in CA1 neurons by pairing 1 Hz presynaptic stimulation of the Schaffer collaterals with moderate postsynaptic depolarization (−40 mV), as previously described [40]. As shown in Figure S5, LTD induction produced a similar decrease in AMPAR-mediated responses in both FGL-treated and untreated neurons. Therefore, we conclude that the enhancement of synaptic plasticity produced by FGL is specific for synaptic potentiation.

It has been reported that NMDARs with different subunit composition play specific roles in different forms of synaptic plasticity, although the details of this specificity are still under debate [41],[42]. As an initial attempt to test whether FGL may alter the subunit composition of NMDARs, we evaluated the kinetics of NMDAR-mediated responses, since NR2A- and NR2B-containing NMDARs display distinct decay time constants [43]. As shown in Figure S6, NMDA responses from FGL-treated neurons displayed faster decay kinetics than those from untreated neurons. However, this effect was not blocked by incubation with chelerythrine to inhibit the PKC pathway (Figure S6, “FGL+Chel”). Therefore, even if FGL alters NMDAR subunit composition, this effect does not appear to be mechanistically linked to the enhancement of LTP and AMPAR synaptic delivery, which require PKC activity. Therefore, we have not pursued this effect any further.

### FGL Does Not Alter Spine Structural Plasticity

Synaptic potentiation has been shown to be accompanied by an increase in spine size and recruitment of polymerized actin into the spine head [44]. Therefore, we tested whether FGL enhances this form of structural plasticity as it enhances synaptic potentiation. To this end, we expressed GFP-actin in organotypic hippocampal slices, and then induced LTP using a pharmacological approach that allows us to maximize the number of synapses undergoing plasticity, while mimicking biochemical and electrophysiological properties of electrically induced LTP [45]. As shown in Figure S7, LTP induction led to an increase in actin-GFP recruitment into spines, which was similar in extent between control and FGL-treated spines (these experiments were analyzed blind with respect to the treatment the slices had received). Therefore, FGL treatment enhances synaptic potentiation, without altering the capacity of spines to undergo structural plasticity, at least as monitored by actin recruitment into the spine head.

### Persistent Activation of Signaling Pathways Upon FGL Treatment

The experiments above indicate that the enhancing effect of FGL on synaptic plasticity does not require the continuous presence of FGL because it can last at least 24 h after FGL removal. This is also consistent with the behavioral effects of FGL treatment, which were manifested 2 d after FGL application (Figure 2). Therefore, we sought long-lasting biochemical signatures of FGL treatment by preparing whole-cell extracts from hippocampal slices at different times after adding FGL to the culture medium, and after its removal 24 h later. We then used Western blot to evaluate three key events linked to upstream FGL action and downstream LTP-related signaling: (i) PKC activation (presumably triggered directly by FGL upon FGFR activation and PLCγ phosphorylation; Figure 1D), (ii) CaMKII phosphorylation (downstream from NMDAR opening and key mediator of LTP expression [46]), and (iii) GluA1 Ser^831^ phosphorylation (triggered during LTP upon CaMKII activation [47] but also catalyzed by PKC [48]).

**Figure 7:**
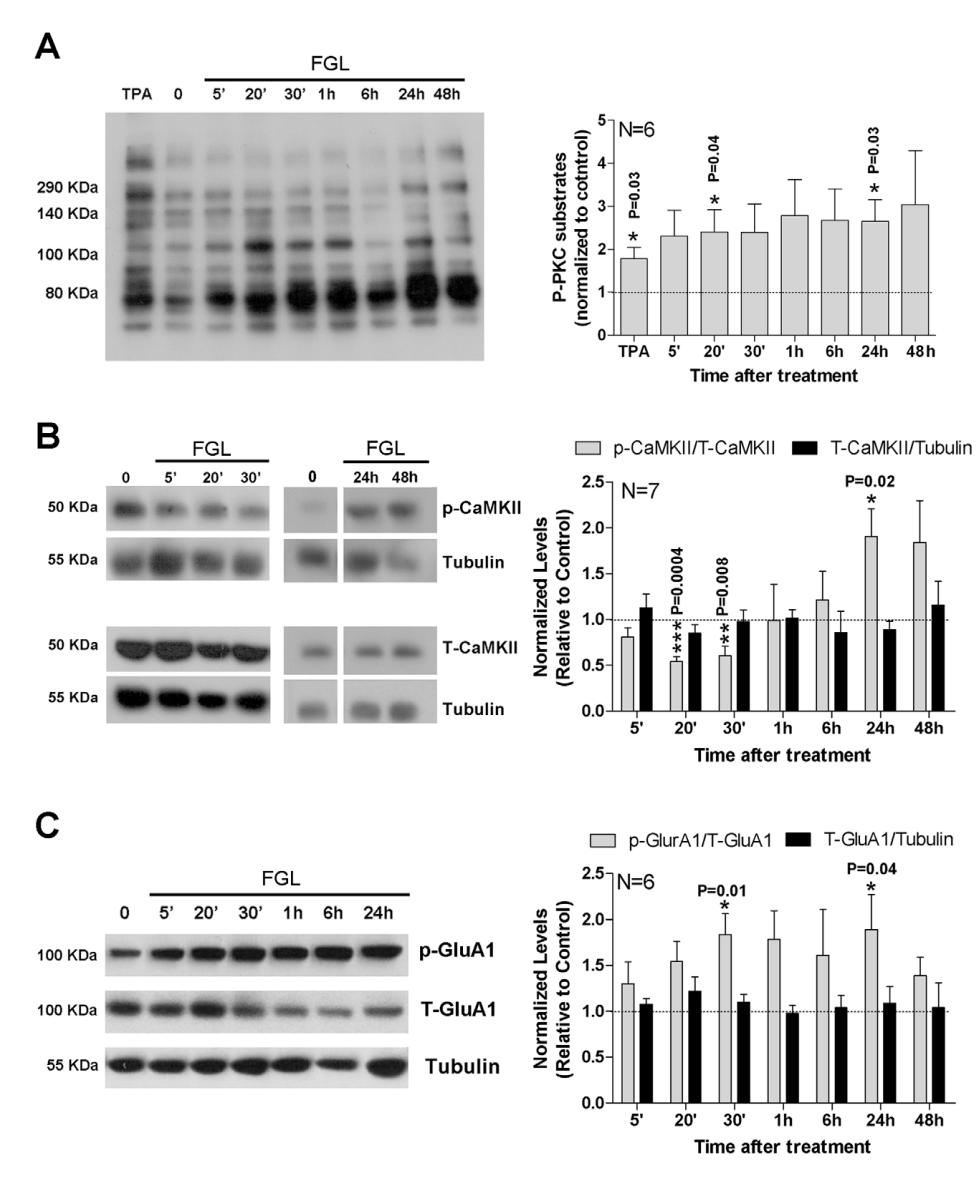
FGL-triggered persistent activation of signaling pathways. (A) Left: Western blot of hippocampal extracts treated with TPA (12-*O*-tetradecanoylphorbol-13-acetate; PKC activator that served as a positive control), untreated (“0”), and treated with FGL at different time-points after FGL application. The primary antibody detects phosphorylation of endogenous proteins at PKC substrate motifs (phospho-(Ser) PKC substrate). Right: Quantification of Western blots similar to the one shown on the left, by calculating the combined intensity from all bands in each lane. *N*, number of independent experiments. The *p* values were determined with the Mann-Whitney test. (B, C) Left: Western blot of hippocampal extracts treated with FGL at different time-points after FGL application and untreated (“0”). The primary antibodies detected phosphorylated CaMKII at Thr286 (p-CaMKII) and total levels of CaMKII (T-CaMKII) (B), or phospho-GluA1 (P-S831) and total GluA1 (C). Tubulin was used as a loading control. Right: Quantification of Western blots similar to the ones shown on the left. *N*, number of independent experiments. The *p* values were determined using the Mann-Whitney test.

To evaluate global PKC activation, we monitored the phosphorylation of multiple PKC substrates with an antibody that recognizes a phospho-Ser PKC substrate motif. Phosphorylation of CaMKII at Thr^286^ and GluA1 at Ser^831^ was monitored with the corresponding phospho-specific antibodies (see Materials and Methods). As shown in Figure 7A, FGL application led to rapid (5–20 min) upregulation of the PKC pathway, which was expected from the activation of FGFR and PLCγ (Figure 1D). Moreover, this pathway remained activated throughout the FGL treatment and 24 h after its removal (Figure 7A, the “48 h” column represents slices treated with FGL for 24 h and then transferred to fresh medium without FGL for 24 h more). Interestingly, CaMKII activation (monitored by Thr^296^ phosphorylation) was transiently decreased by FGL (20–30 min after application), but it showed a gradual upregulation at late time-points, including 24 h after FGL removal (Figure 7B, gray columns). Finally, GluA1 phosphorylation at Ser^831^ was induced early after the addition of FGL but also persisted by the end of the time course (Figure 7C, gray columns). The total levels of CaMKII and GluA1 did not significantly change during or after FGL application (Figure 7B–C, black columns).

These results strongly suggest that FGL initiates signaling mechanisms that outlast the initial triggering events. These mechanisms may be related to a sustained enhanced synaptic activity in the slices, as a consequence of GluA1 delivery.

### Cognitive and Synaptic Effects of FGL Share the PKC Pathway

**Figure 8:**
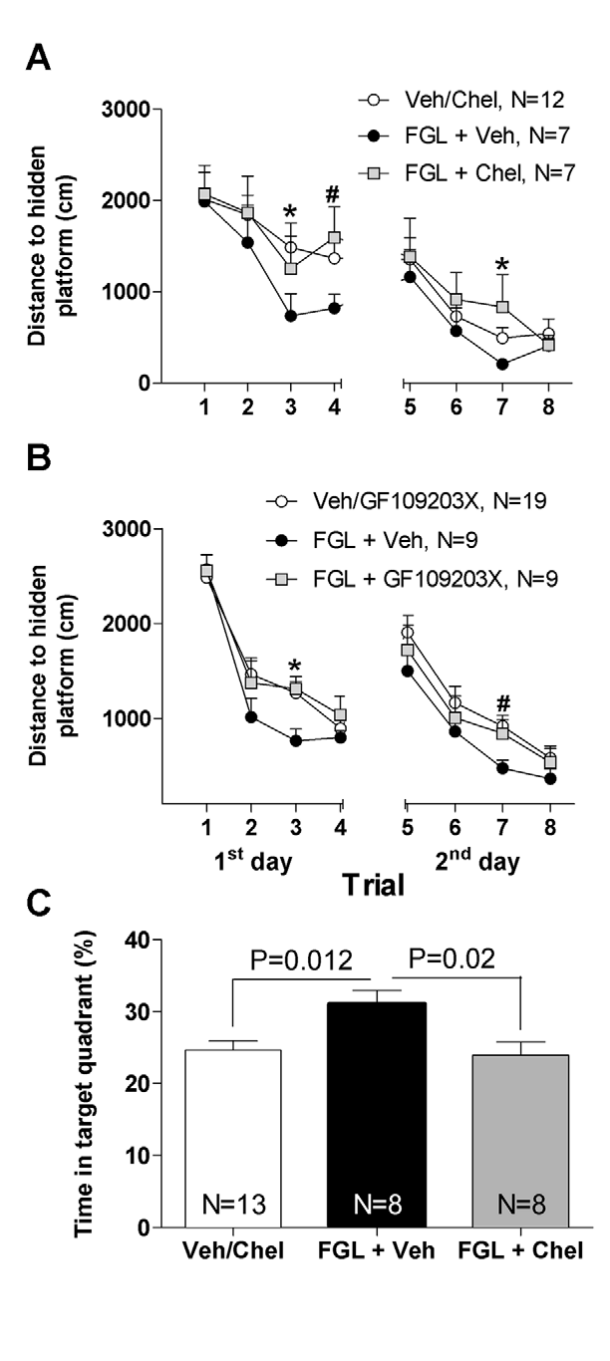
FGL-induced enhanced cognition depends on PKC activity. (A, B) Mean distances traveled to find the hidden platform in the Morris water maze are represented for control rats (white circles), FGL-treated rats (black circles), and rats treated with FGL and the PKC inhibitor (grey squares; A, chelerythrine; B, GF109203X), over the 2 training days (four trials each). *N*, number of animals. Statistical significance was analyzed with repeated-measures ANOVA followed by Bonferroni's post hoc test for individual trials. A: *p<0.05, FGL+Veh compared to FGL+Chel and Veh/Chel groups. #*p*<0.05, FGL+Veh compared to FGL+Chel but not compared to Veh/Chel. B: *p<0.05, FGL+Veh compared to FGL+GF109203X and Veh/GF109203X groups. #*p*<0.05, FGL+Veh compared to Veh/GF109203X but not compared to FGL+GF109203X. (C) Probe test. Average time spent in the target quadrant of the Morris water maze (where the hidden platform had been present during training) for control rats (white column), FGL-treated rats (black column), or rats treated with FGL plus chelerythrine (grey column). Statistical significance was calculated with Bonferroni's post hoc test.

We showed that enhanced synaptic delivery of AMPARs and LTP are induced by FGL in a PKC-dependent manner. We then hypothesized that if facilitated AMPAR synaptic incorporation and LTP contribute to the enhanced cognitive effects of FGL, then blocking the PKC pathway should also block the effects of FGL on learning. To test this point, rats were stereotactically implanted with a double-cannula into the lateral cerebral ventricles. They were then divided into four experimental groups (*n* = 10–14/group) according to treatment (total administered volume, 5 µl): (i) vehicle (artificial CSF [ACSF]), (ii) vehicle +20 µg FGL, (iii) 20 µg FGL +2.5 nmol chelerythrine, and (iv) vehicle +2.5 nmol chelerythrine. Drugs were injected 5 d, 3 d, and 1 d before training. The experimenter who trained the rats was blind to treatment. No side effects were observed following any of the treatments. Because performance of vehicle- and chelerythrine-treated rats was similar in this task (*F*_1,10_ = 0.6, *p = *0.4), data from these two control groups were pooled (see average data in Figure S8A). There were significant differences in the learning curves among the remaining groups (repeated measure ANOVA: *F*_2,22_ = 12.79, *p = *0.001). Similar to our observations with subcutaneous FGL administration (Figure 2), FGL-treated rats (FGL+vehicle) outperformed their controls (vehicle) in the water maze throughout the training procedure (Bonferroni's post hoc test: *p*<0.05 for trials 3 and 7; Figure 8A). Notably, FGL did not enhance learning when combined with chelerythrine (FGL+chelerythrine), indicating that the effects of FGL on cognition depend on PKC activity (Bonferroni's post hoc test: *p*<0.05, for the comparison between FGL+vehicle versus FGL+chelerythrine, for trials 3, 4, and 7). Similar blind experiments were separately carried out with the specific inhibitor of classical PKC isoforms GF109203X. Consistent with our experiments with chelerythrine, co-injection of GF109203X (1 nmol in 5 µl) with FGL blocked the enhanced performance produced by the peptide alone (Figure 8B) (vehicle- and GF109203X-treated rats were similar in this task, and these two control groups were pooled; see separate data in Figure S8B). Finally, similar experiments were attempted with MEK and PI3K inhibitors (PD98059 and LY294002, respectively), but these data could not be easily interpreted due to the intrinsic effect of these drugs on the behavioral task (see Figure S9).

FGL has also been shown to enhance memory consolidation when administered immediately after training [12]. We then tested whether the cognitive effects of FGL on memory were also mediated by the same signaling pathway as the synaptic and learning effects. To this end, rats were stereotactically implanted with cannulae as in the previous experiment, but FGL (with or without chelerythrine) was administered immediately after the training session of each day (see Materials and Methods). Spatial memory was then tested 24 h after the second training day. As shown in Figure 8C, FGL was able to enhance memory under these conditions, as previously described. Most importantly, this enhancement was blocked when FGL was co-administered with chelerythrine, indicating that PKC is also required for FGL-induced memory enhancement.

In conclusion, these behavioral experiments testing the effect of FGL on both learning and memory indicated that enhanced cognition depends on the PKC pathway, similarly to enhanced AMPAR synaptic delivery and LTP. These results strongly suggest that synaptic and cognitive effects of FGL are mechanistically linked.

## Discussion

In this work, we have described a specific molecular mechanism and signaling pathway by which the NCAM mimetic peptide FGL produces a long-lasting enhancement of synaptic plasticity that leads to improved spatial learning and memory. We propose that the facilitation of AMPAR synaptic delivery during learning-induced plasticity events underlies the enhanced cognition produced by FGL. Interestingly, these effects were persistent, and outlasted the initial FGL stimulus. These conclusions are based on three major lines of evidence. First, we showed that FGL produces a long-lasting potentiation of synaptic transmission in hippocampal slices, based on the facilitated synaptic delivery of AMPARs upon NMDAR activation. Second, we found that synapses remain “sensitized” for further LTP induction long after FGL is cleared from the system. Third, both the synaptic and behavioral effects of FGL are based on a long-lasting increase in PKC activity, which is accompanied by a persistent activation of the CaMKII pathway.

A particular novelty of this work relies on linking a direct synaptic modification (i.e., facilitation of AMPAR synaptic delivery) with a high-order cognitive effect (i.e., enhanced spatial learning). Importantly, FGL is acting as a facilitator, rather than a direct trigger, for AMPAR synaptic insertion. That is, FGL-induced potentiation still remains activity-dependent, because it requires NMDAR and CaMKII activation. In fact, it appears that FGL is sensitizing the “classical” LTP pathway, because spontaneous neuronal activity is then able to trigger AMPAR delivery and stabilize synaptic potentiation. This mechanism differs significantly from other neurotrophin-related synaptic modulators, such as BDNF or tumor necrosis factor-α (TNFα), which trigger AMPAR synaptic delivery while bypassing standard LTP signaling [49],[50]. This is a significant distinction, because an effective cognitive enhancer may be expected to facilitate synaptic plasticity events, rather than provoke them in a manner unrelated to ongoing circuit activity. We also want to point out that FGL is acting as a synaptic and cognitive enhancer over physiological levels. That is, FGL treatment increases the extent of synaptic potentiation in naive hippocampal slices, and similarly, it enhances the potential for spatial learning in healthy, young adult rats. These results emphasize the notion that synaptic and cognitive mechanisms can be tuned to operate above normal physiological parameters.

From a mechanistic perspective, it is interesting that FGL produces a persistent enhancement of synaptic plasticity and learning. Thus, we found that a transient activation of FGFR-NCAM signaling by FGL results in long-lasting activation of the PKC and CaMKII pathways, accompanied by enhanced LTP and spatial learning observed 24–48 h after the removal of FGL. We have also determined that the establishment of this persistent enhancement requires PKC activity during the action of FGL. In a sense, this sensitized state produced by FGL is reminiscent of what has been termed *metaplasticity* (i.e., a persistent modification that alters the ability of the synapse to undergo further plasticity events [51]). There is a wide variety of mechanisms that may shift the sensitivity to synaptic plasticity induction, ranging from structural alterations in the extracellular matrix [52] to changes in NMDAR subunit composition [53] or metabotropic glutamate receptor activation [54]. With regard to FGL and the subsequent activation of FGFR-NCAM signaling, we determined that the functional synaptic changes are not accompanied by detectable alterations in morphology of dendritic spines, ultrastructural synaptic organization, or presynaptic release properties (this is in contrast with the reported effects of FGL on synaptogenesis and presynaptic function on primary neuronal cultures [12]). On the other hand, we established that PKC activity is required to reach this sensitized state, followed by a long-lasting increase in CaMKII activity and AMPAR phosphorylation at GluA1 Ser^831^. Interestingly, these changes do not saturate (or occlude) further LTP expression. In fact, the potential for LTP expression is actually enhanced after FGL treatment. These considerations lead us to propose a model in which FGL-triggered FGFR-NCAM signaling acts on upstream targets that facilitate LTP induction. Subsequently, ongoing neuronal activity will be more likely to activate NMDARs and CaMKII, leading to enhanced synaptic delivery of AMPARs and potentiation of synaptic responses. This strengthening of excitatory connections may in turn facilitate the induction of further LTP-like events, resulting in the observed long-lasting increase in PKC and CaMKII activation, and AMPAR phosphorylation. These synaptic and biochemical changes will persist, as long as there is ongoing neuronal activity in the circuit.

In conclusion, the present study has provided mechanistic insights into the synaptic events and molecular cascades that mediate the enhanced cognitive function produced by a pharmacological mimetic of cell adhesion-growth factor signaling.

## Materials and Methods

For a more detailed description of the methods, see Text S1.

### FGL Preparation

The pentadecapeptide FGL (EVYVVAENQQGKSKA), corresponding to residues E681 to A695 of the second fibronectin domain of NCAM (Figure 1A), was synthesized using 9-fluorenylmethoxycarbonyl (Fmoc) solid phase peptide synthesis methodology. FGL was used in dimeric form by linking two monomers through their *N*-terminal ends with iminodiacetic acid (*N*-carboxymethyl)-glycine (Polypeptide Laboratories, Hillerød, Denmark). This dimeric design binds and brings together two FGFR molecules, which is crucial for receptor phosphorylation and downstream signaling [55]. The *C*-terminal ends of the peptides were amidated. The peptide was at least 85% pure as estimated by high-performance liquid chromatograhy.

### In Vivo FGL Treatments

#### Subcutaneous FGL administration

Male Wistar rats (Harlan, Barcelona, Spain) between 8 and 9 wk of age (weighing 240–260 g) were subcutaneously injected with FGL (6.6 mg/kg body weight) or vehicle (0.9% NaCl) alone, 5 and 2 d before training began (for behavioral testing) or before sacrifice (for morphological experiments). For the biochemical experiments (FGFR1 and TrkB phosphorylation), a single FGL/vehicle injection was administered 1 h before sacrifice at a dose of 2.2, 6.6, or 8.8 mg/kg body weight. No side effects were observed following these treatments.

#### Intracerebroventricular injection procedure

Approximately 2 wk after arrival, rats were prepared for intracerebroventricular cannulation. Rats were anesthetized intraperitoneally with 2,2,2-tribromomethanol (250 mg/kg, Aldrich, Milwaukee, WI). A 22-gauge double-guide injection cannula (C313G, Plastic One, Roanoke, VA) fitted with a removable dummy cannula was stereotactically implanted into the lateral cerebral ventricles 1.3 mm posterior, 1.6 mm lateral, and 3.5 mm ventral, and fixed with two screws in the skull using dental cement (Duralay 2244, Reliance). At least 12 d were allowed for recovery from surgery before any behavioral test. After surgery, the animals were housed in pairs, and their body weights were monitored.

Protein kinase inhibitors were prepared in an aCSF vehicle solution, with 1% DMSO (chelerythrine), 20% cyclodextrane (GF109203X), or 1% DMSO plus 20% cyclodextrane (PD98059 and LY294002). FGL was always prepared in the same vehicle solution as the kinase inhibitors present in the corresponding experiment. A flexible swivel attached to the rat allowed the peptide to be administered while the animal was conscious and freely moving in a cage. For learning experiments (Figures 2 and 8A, B), FGL was applied 5, 3, and 1 d before water maze training. For memory experiments (Figure 8C), FGL was infused immediately after each training session. The dummy cannula was removed and replaced with a 28-gauge infusion cannula (model C313I, Plastics One, Roanoke, VA) attached to a 2.5 ml Hamilton syringe via polyurethane tubing (fluorinated ethylene propylene, 0.12 mm diameter CMA, Microdialysis AB, Stockholm, Sweden). A microinjection pump (Harvard Apparatus, Cambridge, MA) controlled the delivery of 5 µl of solution through the cannulas at a rate of 1.25 µl/min. The cannulas were left in place for an additional 3 min to allow diffusion of the peptide away from the cannula tip before replacement with the dummy cannula. All rats were habituated to this injection procedure (i.e., no solution was injected) for 2 min daily for 2 d preceding the proper injection on the day of the experiment.

All biosafety procedures and animal care protocols were approved by the bioethics committee from the Consejo Superior de Investigaciones Cientificas (CSIC), and were carried out according to the guidelines set out in the European Community Council Directives (86/609/EEC).

### Behavior

Morris water maze spatial learning was performed under mild training conditions to assess any modulation of learning related to FGL administration. For details, see Text S1.

### Biochemistry

Protein extracts from hippocampal slices were prepared in 10 mM HEPES, 150 mM NaCl, 10 mM EDTA, 0.1 mM phenylmethanesulphonylfluoride (PMSF), 2 µg/ml chymostatin, 2 µg/ml leupeptin, 2 µg/ml antipain, 2 µg/ml pepstatin, 10 mM NaF, 1 µM microcystin LR, 0.5 µM calyculin A, and 1% Triton X-100. Western blots were developed with chemiluminescence (SuperSignal Kit; Pierce, Rockford, IL) and quantified using a densitometric scanning under linear exposure conditions.

#### Antibodies

Western blot or ELISA analyses were conducted with anti-phospho-FGFR-1 (pYpY653/654, Biosource International), anti-phospho-TrkB (Abcam), anti-phospho-GluA1 (P-S831 and P-S845, Upstate Biotechnologies), anti-total GluA1 (Abcam), anti-phospho-CaMKII (Thr286, Chemicon), and anti-total CaMKIIα (Sigma). PLCγ1, phospho-PLCγ1 (Tyr783), FRS2α, phospho-FRS2α (Tyr436), ShcA, phospho-ShcA (Tyr239/240), MARCKS and phospho-MARCKS (Ser152/156) antibodies were obtained from Cell Signalling. Strep II tag antibody was from QIAGEN (Copenhagen, Denmark). Agarose-coupled anti-phosphotyrosine antibody (4G10-AC) was from Upstate Biotechnologies.

### Fluorescence Microscopy

Rats (*n* = 8/group) were perfused with 4% paraformaldehyde (pH 7.4). Coronal sections (150 µm) were cut on a vibratome. Cells in the hippocampus were individually injected with Lucifer Yellow. Imaging was performed on a Leica laser scanning multispectral confocal microscope (TCS SP5) using an argon laser. After acquisition, the stacks were processed with a 3-dimensional blind deconvolution algorithm (Autodeblur; Autoquant, Media Cybernetics) to reduce the out-of-focus light (see example in Figure 3F). The 3-dimensional image processing software IMARIS 5.0 (Bitplane AG, Zurich, Switzerland) was used to measure the spine head volume and neck length (see also Text S1) [30]. For spine density analysis, dendrites were traced with the Neurolucida 7.1 computerized data collection system. Spine density was automatically calculated by dividing the number of spines on a dendrite by the dendrite length. See Text S1 for further details.

### Electron Microscopy

Sections adjacent to those used for the intracellular injections were processed for electron microscopy. These sections were embedded in Araldite and studied using a correlative light and electron microscopic method (described in detail in Text S1). The two major morphological types of cortical synapses, namely asymmetrical and symmetrical types [29], were clearly identified in the analyzed tissue. The synapses in which the synaptic cleft and associated membrane densities could not be visualized clearly because of the oblique plane of the section were considered uncharacterized synapses. Synaptic density per unit area (NA) was estimated from electron microscope samples of neuropil of the stratum radiatum of CA1. The density of synapses per unit volume of the neuropil was calculated using the formula *NV* = *NA*/*d*, in which *NA* is the number of synaptic profiles per unit area and *d* is the average cross-sectional length of synaptic junctions (for a detailed description, see [29]).

### Organotypic Cultures and FGL Treatment

Hippocampal slice cultures were prepared from postnatal day 5–6 rats [56]. After 4–8 d in culture, FGL was applied to the culture medium (10 µg/ml), and the medium was refreshed, without FGL, 24 h later. Electrophysiological recordings were performed 2 d after beginning the FGL treatment.

### Electrophysiology

Voltage-clamp whole-cell recordings were obtained from CA1 pyramidal neurons under visual guidance using fluorescence and transmitted light illumination. For details regarding the internal and external solutions, see Text S1. Synaptic AMPAR-mediated responses were acquired at −60 mV. NMDAR responses were recorded at +40 mV at a latency at which AMPAR responses were fully decayed (60 ms after stimulation). In both cases, 100 µM picrotoxin was present in the external solution. GABA_A_ receptor responses were recorded at 0 mV in the presence of 100 µM AP5 in the external solution. For the rectification studies, GluA1-GFP was expressed in CA1 neurons for 60 h, and AMPAR responses were recorded at −60 mV and +40 mV in the presence of 0.1 mM AP5 in the external solution and 0.1 mM spermine in the internal solution. Because only CA1 cells (and not CA3 cells) were infected, this configuration ensured that GluA1-GFP was always expressed exclusively in the postsynaptic cell. When specific kinase inhibitors were used, the inhibitor was added to the culture medium 1 h before the addition of FGL. The medium was refreshed 1 d later with the same inhibitor but without FGL. Electrophysiological recordings were performed 1 d later in the presence of the kinase inhibitor (with the exception of chelerythrine, which was neither added to the medium upon refreshing nor present during the recordings). LTP was induced using a pairing protocol by stimulating Schaffer collateral fibers at 3 Hz for 1.5 min while depolarizing the postsynaptic cell at 0 mV. Whole-cell recordings were made with a Multiclamp 700A amplifier (Axon Instruments).

### Statistical Analysis

For the behavioral results, the data were analyzed with SPSS version 11 (Chicago, IL, USA). Morris water maze training data were analyzed across trials with one-way analysis of variance (ANOVA), with the training trial as the repeated measure, followed by Bonferroni's post hoc test when appropriate. For the morphological parameters, the data were averaged to obtain the cell mean, and the neurons from each animal were averaged for the animal mean. Normality was tested using the Kolmogorov-Smirnov test. Because both the spine density and spine morphology values had Gaussian distributions, we used a two-tail unpaired *t* test to assess the overall effect. When comparing mean electrophysiological values, statistical significance was determined by the Mann-Whitney test (unless indicated differently) if only two distributions were compared or by ANOVA followed by the Kruskal-Wallis test if multiple distributions were analyzed.

## Supporting Information

Figure S1 Unaltered spine neck length after FGL treatment. (A) Representative confocal projection image of CA1 pyramidal neurons. Bar = 150 µm. (B) Representative high magnification confocal projection image of apical dendrites to illustrate dendritic spines. Bar = 1.5 µm. (C) Quantification of spine neck length measured three-dimensionally. *N*, number of rats. (D) Cumulative frequency of neck length values. *N*, number of spines.
(TIF)

Figure S2 Effects of FGL on passive membrane properties of CA1 hippocampal neurons. (A–B) Application of FGL to cultured hippocampal slices did not alter input resistance (a) or holding current (b) when compared with untreated control neurons. *N*, number of cells.
(TIF)

Figure S3 Chelerythrine inhibition of PKC activity on MARCKS (Myristoylated Alanine-Rich C-Kinase Substrate). (A) Left panels. Representative confocal images of BHK cells transfected with GFP-MARCKS under basal conditions (“no treatment”) or 20 s after application of the PKC activator 12-O-tetradecanoylphorbol-13-acetate (“TPA,” 0.1 µM). Right panels. Similar experiments were carried out in the presence of 10 µM chelerythrine. (B) Quantification of GFP-MARCKS fluorescence intensity ratios between the cell plasma membrane and the cytosol from two independent experiments as the one shown in (A). N represents number of cells. (C) Western blot of hippocampal extracts from slices treated for 30 min with TPA (0.5 µM), chelerythrine (10 µM), or a combination of both, as indicated. Control slices were treated with vehicle (0.1% DMSO). Phosphorylation of MARCKS at the PKC specific sites Ser152/156, total levels of MARCKS, and tubulin (as loading control) were monitored with specific antibodies. (D) Quantification of phosphorylated to total ratios of MARCKS from four independent experiments as the one shown in (C).
(TIF)

Figure S4 FGL facilitates LTP in CA1 neurons. Cumulative frequency distribution of EPSC fold potentiation from the individual LTP experiments plotted in Figure 6D–E. Data are presented for control slices or slices treated with FGL, chelerythrine, or FGL plus chelerythrine, as indicated.
(TIF)

Figure S5 FGL does not alter long-term synaptic depression. Organotypic slice cultures were treated with FGL for 24 h and then transferred to fresh culture medium (without FGL) for an additional 24 h prior to recordings. Control slices were kept in regular culture medium until the recordings. LTD was induced by pairing presynaptic 1 Hz stimulation (300 pulses) with moderate postsynaptic depolarization (−40 mV) (black bar). Inset: sample traces of evoked AMPAR-mediated synaptic responses before (thin line) and after (thick line) LTD induction. *N*, number of cells.
(TIF)

Figure S6 FGL alters NMDAR decay kinetics in a PKC-independent manner. Organotypic slice cultures were treated with FGL (with or without chelerythrine) for 24 h and then transferred to fresh culture medium for an additional 24 h prior to recordings. Control slices were kept in regular culture medium until the recordings. NMDAR-mediated synaptic responses were recorded at +40 mV in the presence of CNQX (AMPAR antagonist). (A) Average trace of NMDAR response normalized to its peak amplitude from untreated (blue), FGL-treated (grey), or FGL plus chelerythrine-treated slices (red). Standard error of the mean is plotted for each trace as thin dashed lines. (B) Average half-decay time (T_1/2_) of NMDA responses from the same data plotted in (A). FGL treatment produces a significant reduction in the half-decay time, which is not blocked by PKC inhibition (chelerythrine).
(TIF)

Figure S7 FGL does not alter structural plasticity of dendritic spines. (A) Representative confocal fluorescence image of dendritic spines expressing actin-GFP before (0′) or at different times after induction of LTP (5′, 20′) from untreated or FGL-treated organotypic slices. Spine heads undergoing plasticity are marked with arrows. LTP was induced using a standard pharmacological protocol (see Text S1). (B) Time-course of actin-GFP fluorescence at individual spine heads before, during (grey shade), and after LTP induction, from images as the one shown in (A). Fluorescence values in the spine head were normalized to the average value in the dendritic shaft (to compensate for ongoing fluorescence bleaching) and expressed relative to the baseline. Analysis was done blind with respect to the treatment the slices had received.
(TIF)

Figure S8 PKC inhibition does not alter spatial learning. Mean distances swam to find the hidden platform in the Morris water maze from vehicle- (white column) and PKC inhibitor-treated rats (gray columns; A, chelerythrine; B, GF109203X), over the eight training trials (four trials per day). *N*, the number of animals.
(TIF)

Figure S9 Spatial learning in the Morris water maze is altered by MEK and PI3K inhibitors. Mean distances traveled to find the hidden platform in the Morris water maze over the 2 training days (four trials each). *N*, number of animals. Rats injected with vehicle, FGL (20 µg), PD98059 (MEK inhibitor, 20 nmol), or LY294002 (PI3K inhibitor, 4.5 nmol), as indicated.
(TIF)

Text S1 Additional experimental procedures are described in this supplementary text.
(DOCX)

## Abbreviations

aCSF: artificial cerebrospinal fluid
AMPA: 2-amino-3-(5-methyl-3-oxo-1,2- oxazol-4-yl)propanoic acid
AMPAR: AMPA receptor
ANOVA: analysis of variance
AP5: (2R)-amino-5-phosphonovaleric acid
BDNF: brain-derived neurotrophic factor
CA1: *cornus ammonis* 1
CA3: *cornus ammonis* 3
CaMKII: Ca^2+^/calmodulin-dependent protein kinase II
CNQX: 6-cyano-7-nitroquinoxaline-2,3-dione
CSF: cerebrospinal fluid
DAB: 3,3′-Diaminobenzidine
DAPI: 4′,6-diamidino-2-phenylindole
DMSO: dimethyl sulfoxide
EDTA: ethylenediaminetetraacetic acid
EGTA: ethyleneglycoltetraacetic acid
ELISA: enzyme-linked immunosorbent assay
EPSC: excitatory postsynaptic current
FGF: fibroblast growth factor
FGFR: fibroblast growth factor receptor
FGL: FG loop peptide, containing F and G strands and the connecting loop in the second F3 module of NCAM
FRS2: FGFR substrate 2
GABA: gamma-aminobutyric acid
HEPES: 4-(2-hydroxyethyl)-1-piperazineethanesulfonic acid
LTP: long-term potentiation
MAPK: mitogen-activated protein kinase
MARCKS: myristoylated alanine-rich C-kinase substrate
MEK: MAPK kinase
NCAM: neural cell adhesion molecule
NMDA: N-Methyl-D-aspartate
NMDAR: NMDA receptor
PB: phosphate buffer
PI3K: phosphoinositide-3-kinase
PKC: protein kinase C
PLC: phospholipase C
PMSF: phenylmethanesulfonylfluoride
PPF: paired pulse facilitation
Shc: Src homologous and collagen
TPA: 12-*O*-tetradecanoylphorbol-13-acetate
