# Supplementary figures and images for: Facilitation of AMPA Receptor Synaptic Delivery as a Molecular Mechanism for Cognitive Enhancement
Source: PLoS Biol. 2012 Feb 21;10(2):e1001262. doi: 10.1371/journal.pbio.1001262 (PMC3283560; doi:10.1371/journal.pbio.1001262)

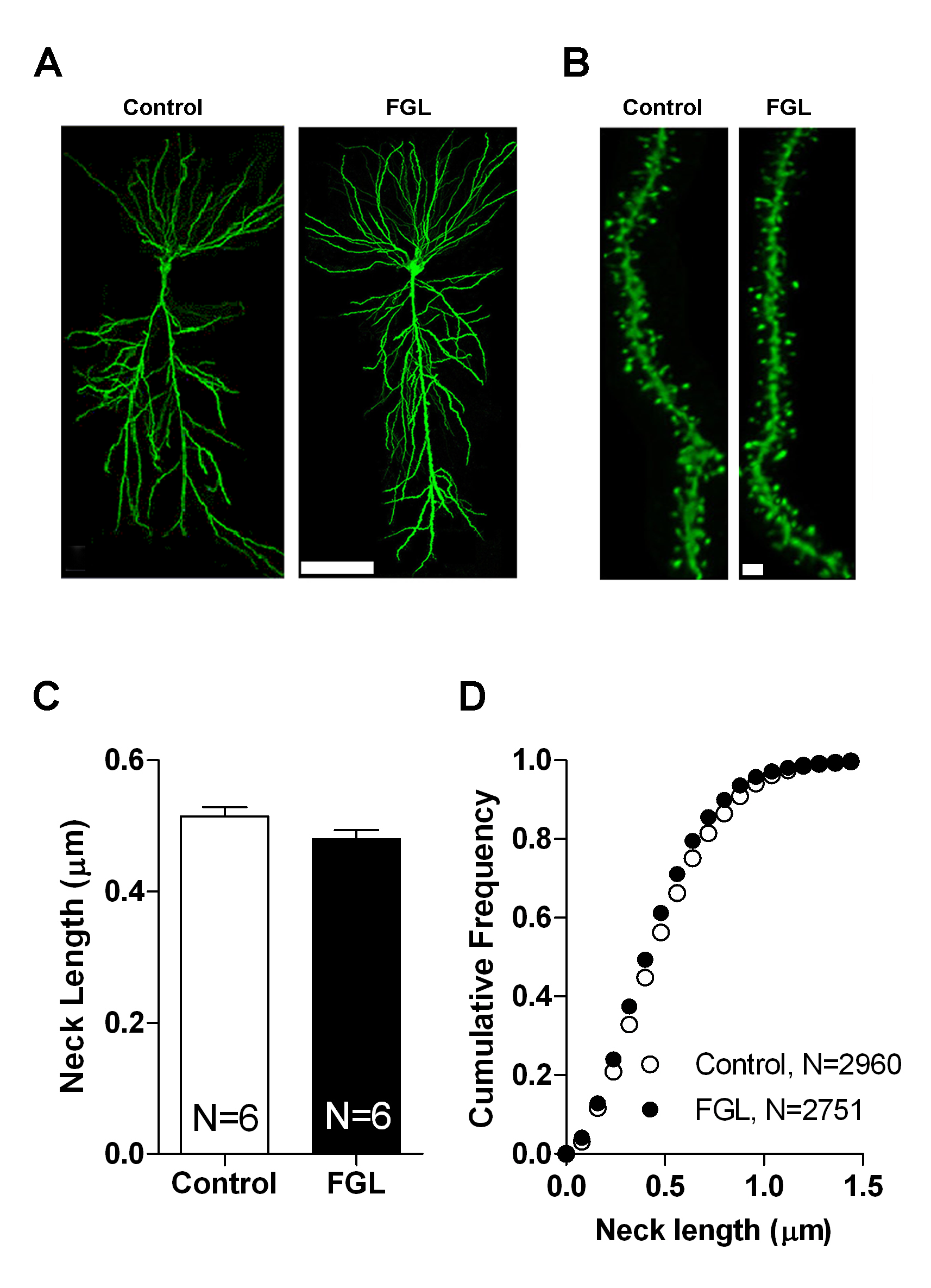

Supplement: Figure S1 — Unaltered spine neck length after FGL treatment. (A) Representative confocal projection image of CA1 pyramidal neurons. Bar = 150 µm. (B) Representative high magnification confocal projection image of apical dendrites to illustrate dendritic spines. Bar = 1.5 µm. (C) Quantification of spine neck length measured three-dimensionally. N, number of rats. (D) Cumulative frequency of neck length values. N, number of spines. (TIF) [file pbio.1001262.s001.tif]

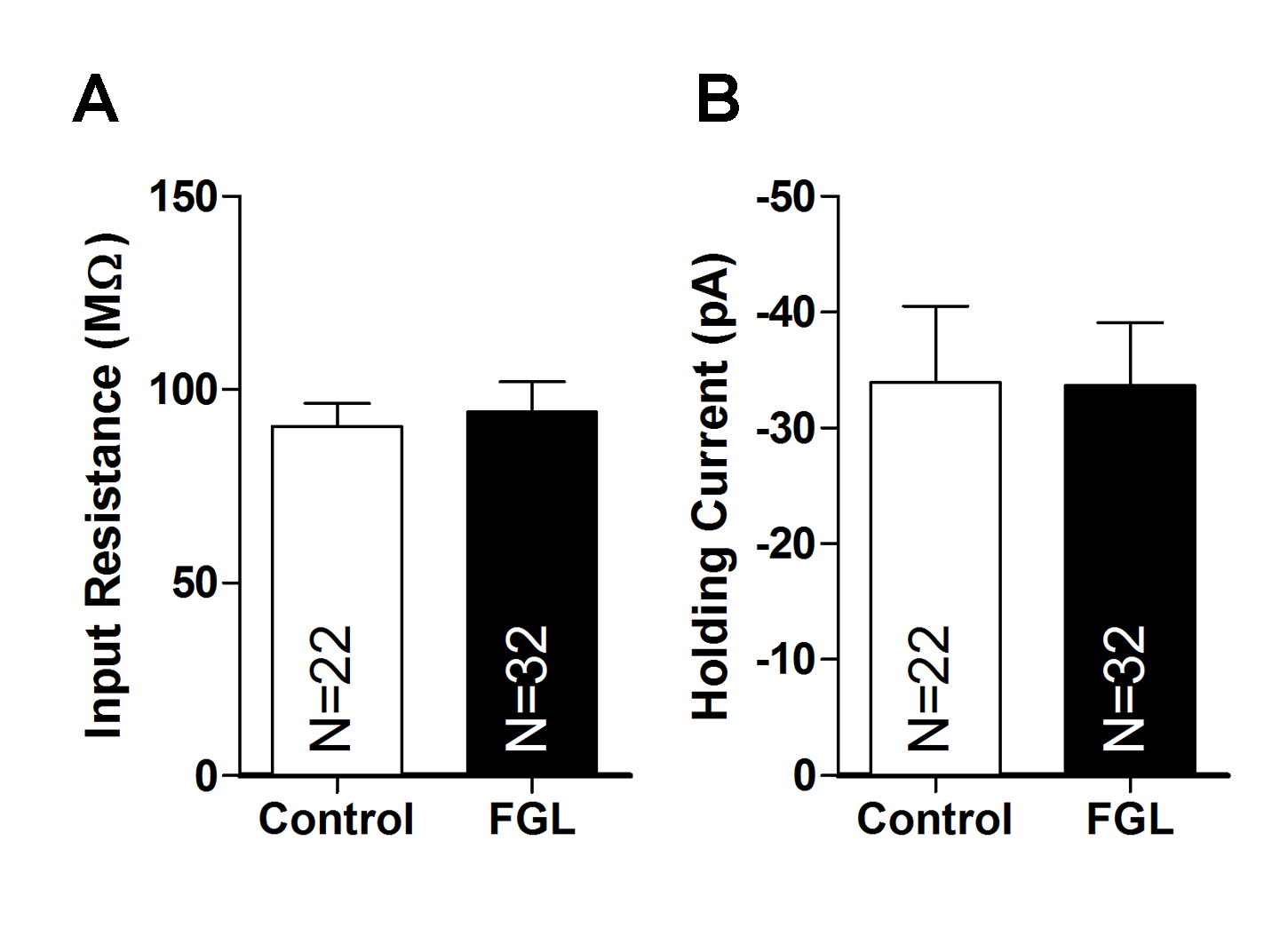

Supplement: Figure S2 — Effects of FGL on passive membrane properties of CA1 hippocampal neurons. (A–B) Application of FGL to cultured hippocampal slices did not alter input resistance (a) or holding current (b) when compared with untreated control neurons. N, number of cells. (TIF) [file pbio.1001262.s002.tif]

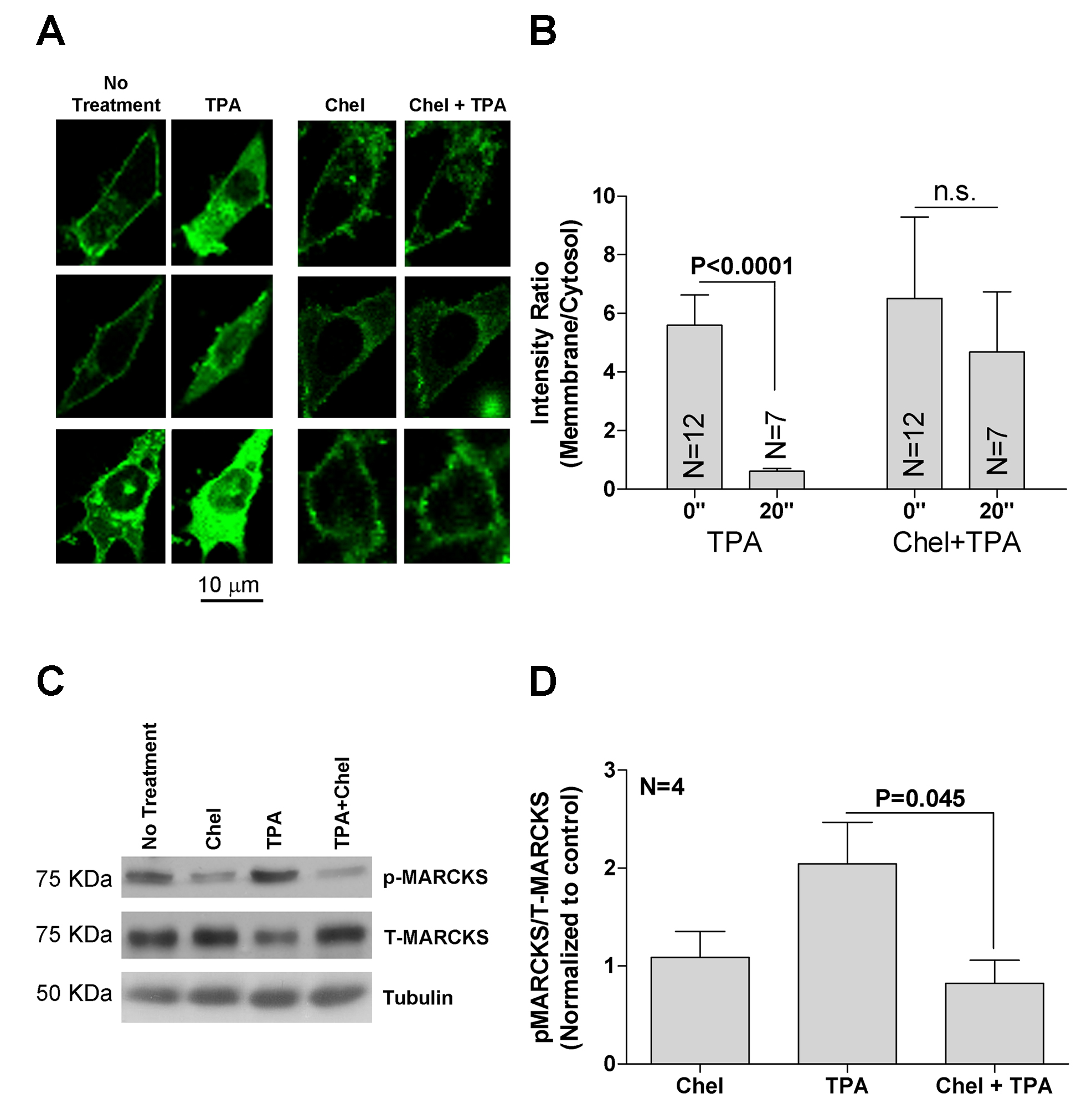

Supplement: Figure S3 — Chelerythrine inhibition of PKC activity on MARCKS (Myristoylated Alanine-Rich C-Kinase Substrate). (A) Left panels. Representative confocal images of BHK cells transfected with GFP-MARCKS under basal conditions (“no treatment”) or 20 s after application of the PKC activator 12-O-tetradecanoylphorbol-13-acetate (“TPA,” 0.1 µM). Right panels. Similar experiments were carried out in the presence of 10 µM chelerythrine. (B) Quantification of GFP-MARCKS fluorescence intensity ratios between the cell plasma membrane and the cytosol from two independent experiments as the one shown in (A). N represents number of cells. (C) Western blot of hippocampal extracts from slices treated for 30 min with TPA (0.5 µM), chelerythrine (10 µM), or a combination of both, as indicated. Control slices were treated with vehicle (0.1% DMSO). Phosphorylation of MARCKS at the PKC specific sites Ser152/156, total levels of MARCKS, and tubulin (as loading control) were monitored with specific antibodies. (D) Quantification of phosphorylated to total ratios of MARCKS from four independent experiments as the one shown in (C). (TIF) [file pbio.1001262.s003.tif]

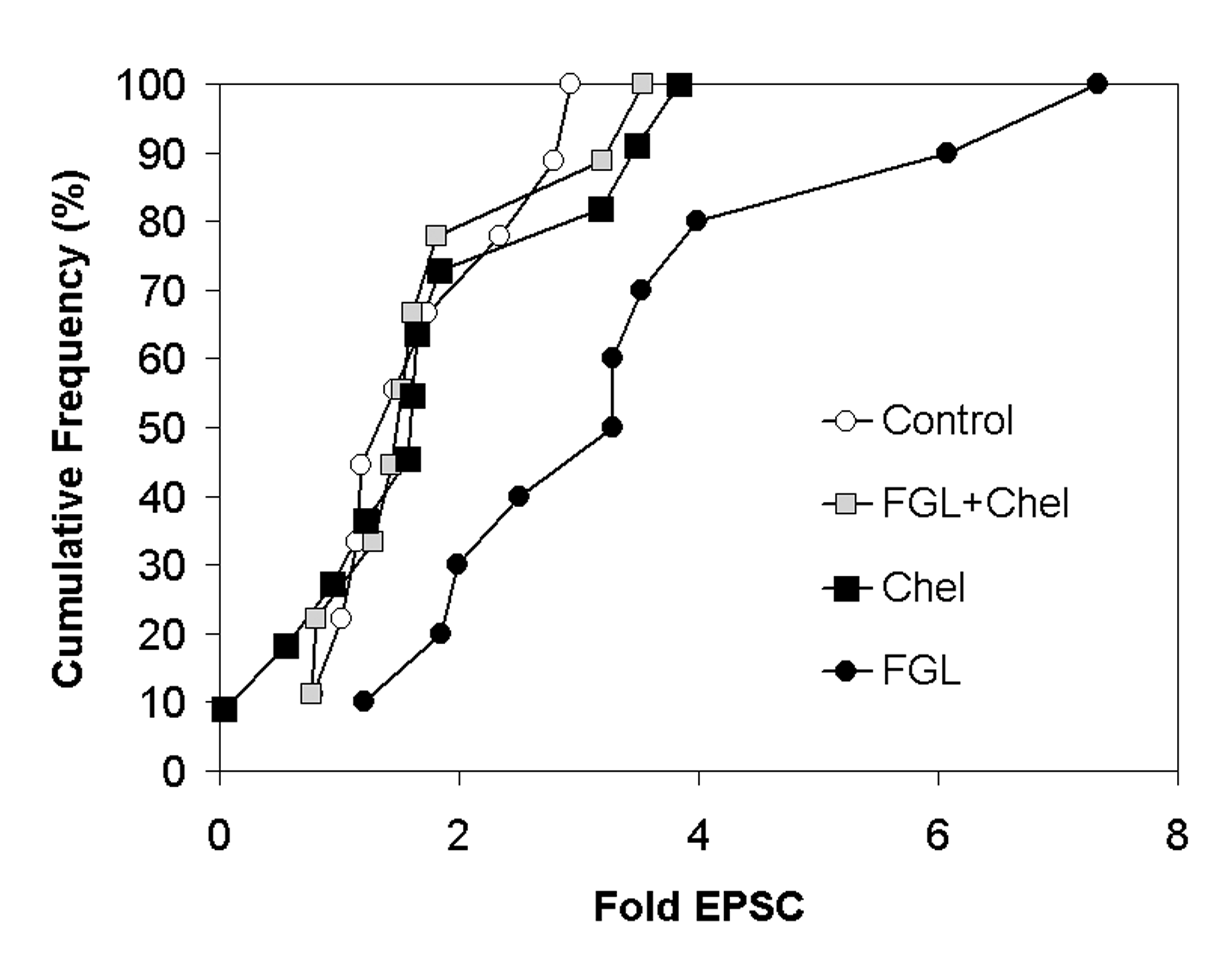

Supplement: Figure S4 — FGL facilitates LTP in CA1 neurons. Cumulative frequency distribution of EPSC fold potentiation from the individual LTP experiments plotted in Figure 6D–E. Data are presented for control slices or slices treated with FGL, chelerythrine, or FGL plus chelerythrine, as indicated. (TIF) [file pbio.1001262.s004.tif]

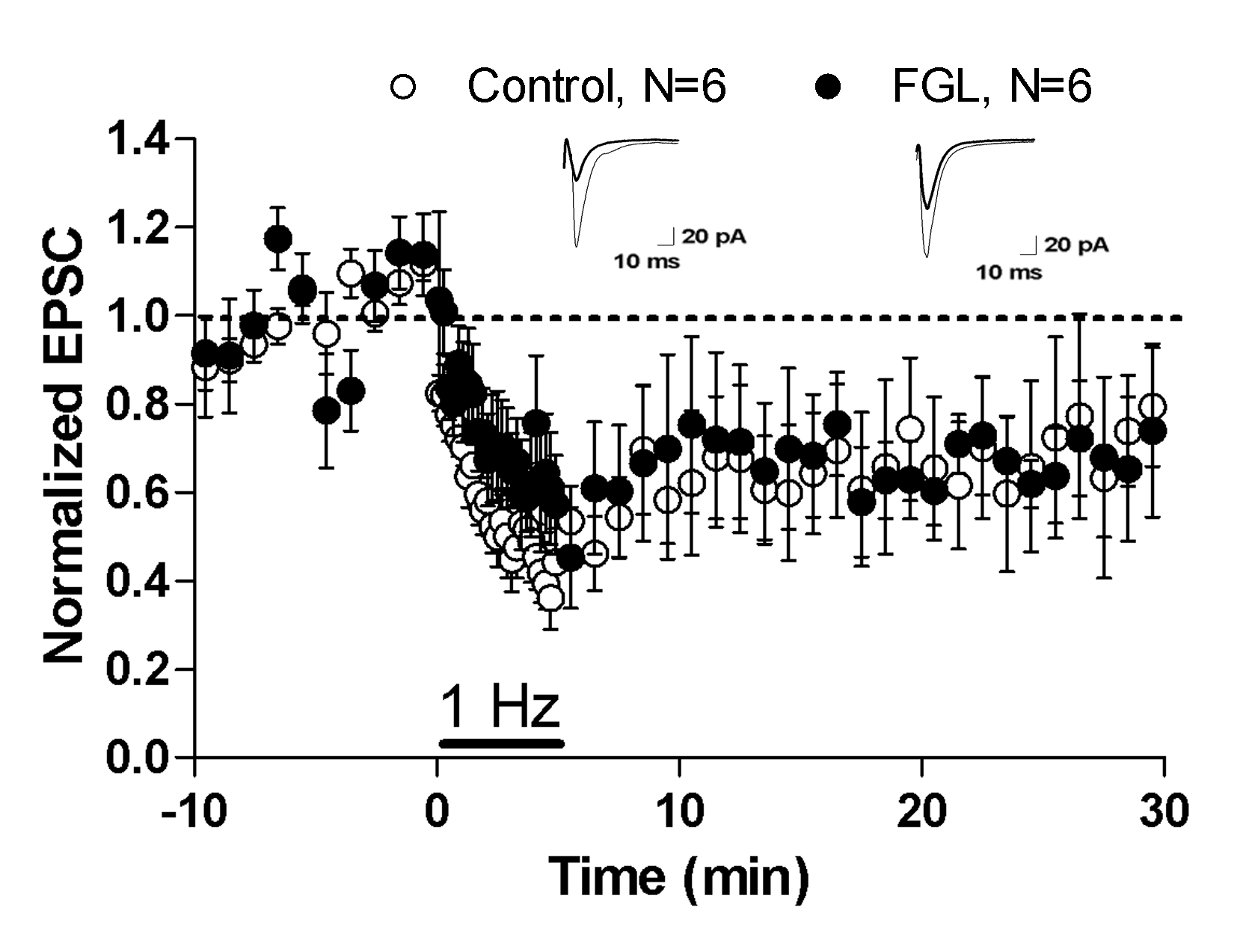

Supplement: Figure S5 — FGL does not alter long-term synaptic depression. Organotypic slice cultures were treated with FGL for 24 h and then transferred to fresh culture medium (without FGL) for an additional 24 h prior to recordings. Control slices were kept in regular culture medium until the recordings. LTD was induced by pairing presynaptic 1 Hz stimulation (300 pulses) with moderate postsynaptic depolarization (−40 mV) (black bar). Inset: sample traces of evoked AMPAR-mediated synaptic responses before (thin line) and after (thick line) LTD induction. N, number of cells. (TIF) [file pbio.1001262.s005.tif]

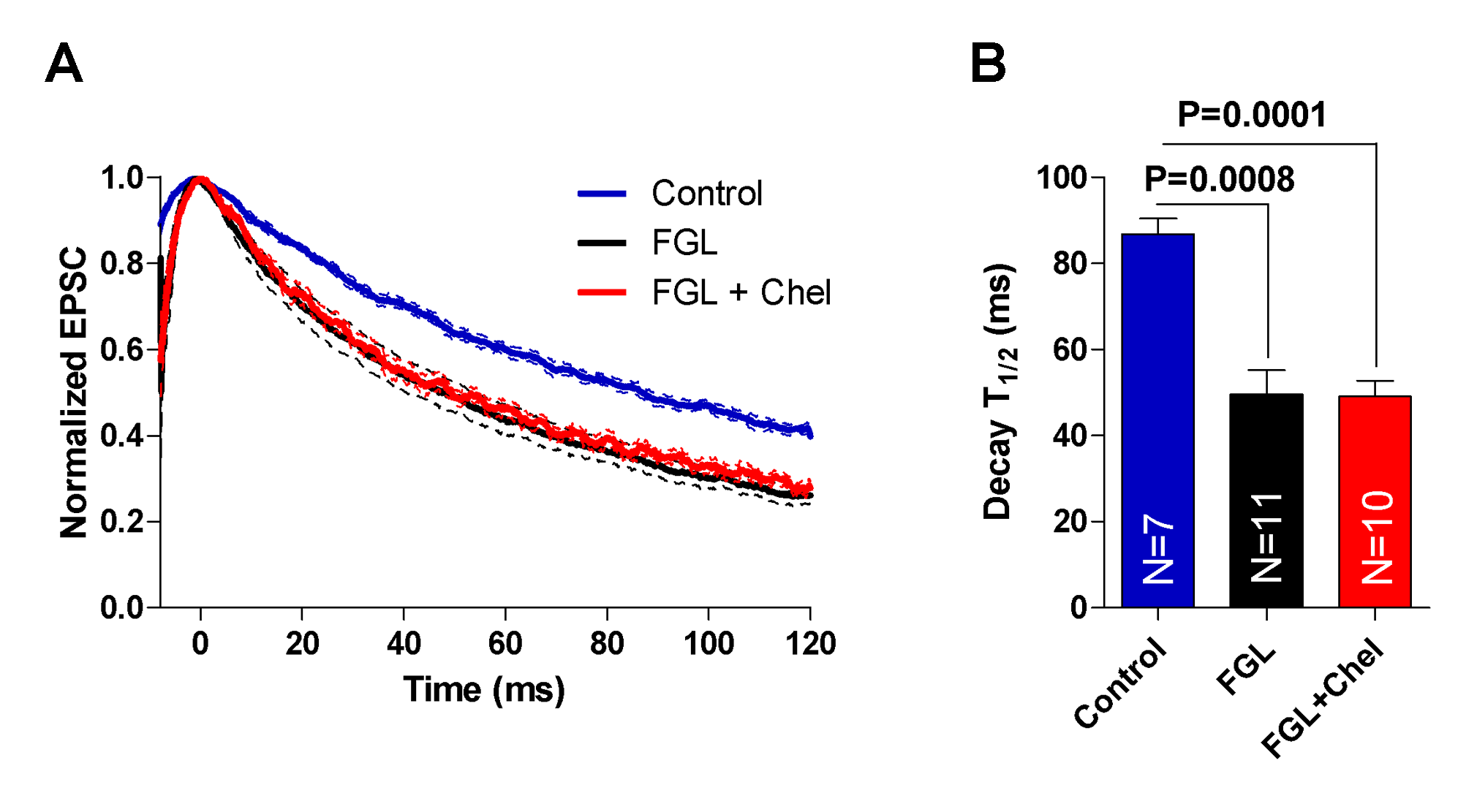

Supplement: Figure S6 — FGL alters NMDAR decay kinetics in a PKC-independent manner. Organotypic slice cultures were treated with FGL (with or without chelerythrine) for 24 h and then transferred to fresh culture medium for an additional 24 h prior to recordings. Control slices were kept in regular culture medium until the recordings. NMDAR-mediated synaptic responses were recorded at +40 mV in the presence of CNQX (AMPAR antagonist). (A) Average trace of NMDAR response normalized to its peak amplitude from untreated (blue), FGL-treated (grey), or FGL plus chelerythrine-treated slices (red). Standard error of the mean is plotted for each trace as thin dashed lines. (B) Average half-decay time (T1/2) of NMDA responses from the same data plotted in (A). FGL treatment produces a significant reduction in the half-decay time, which is not blocked by PKC inhibition (chelerythrine). (TIF) [file pbio.1001262.s006.tif]

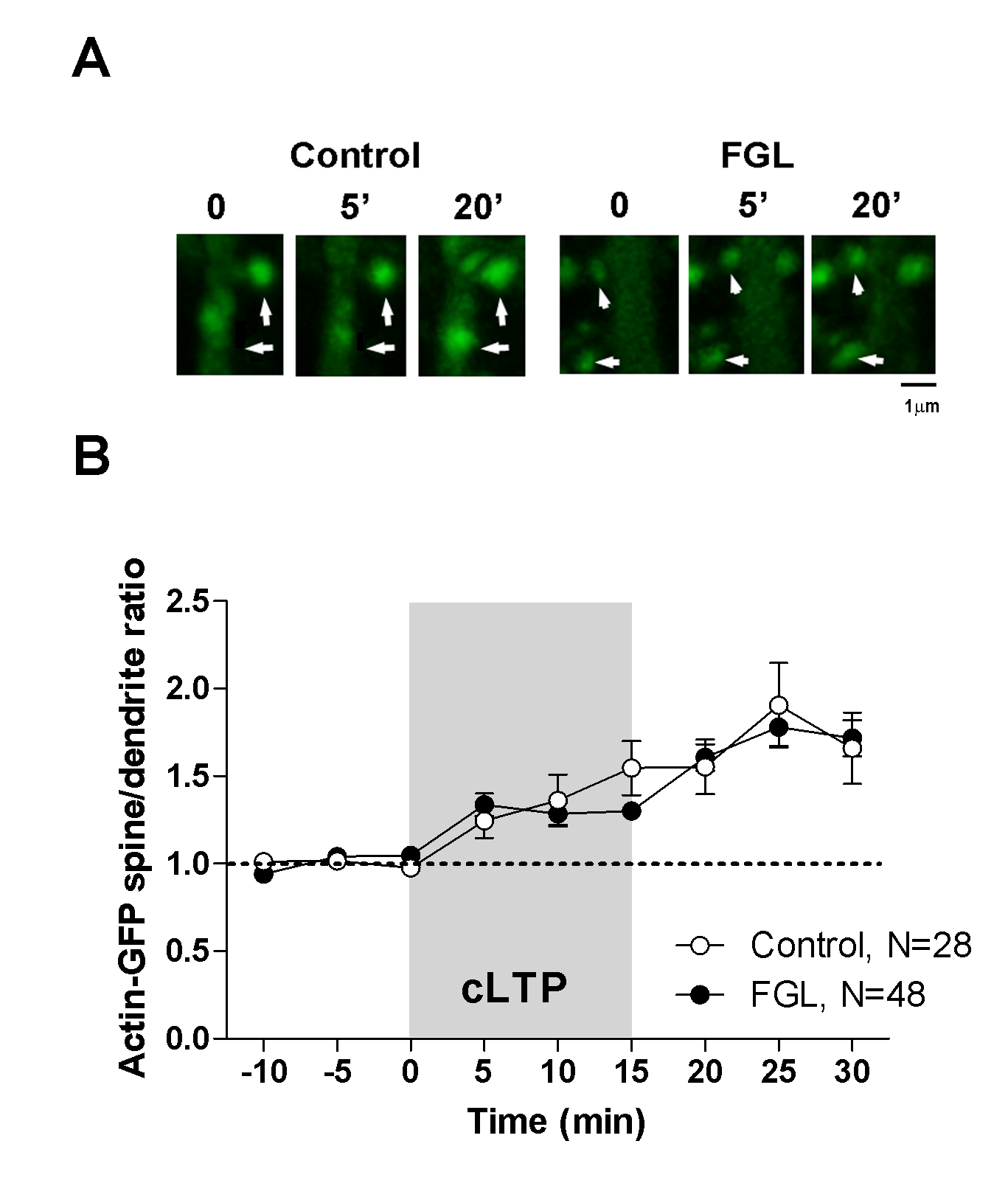

Supplement: Figure S7 — FGL does not alter structural plasticity of dendritic spines. (A) Representative confocal fluorescence image of dendritic spines expressing actin-GFP before (0′) or at different times after induction of LTP (5′, 20′) from untreated or FGL-treated organotypic slices. Spine heads undergoing plasticity are marked with arrows. LTP was induced using a standard pharmacological protocol (see Text S1). (B) Time-course of actin-GFP fluorescence at individual spine heads before, during (grey shade), and after LTP induction, from images as the one shown in (A). Fluorescence values in the spine head were normalized to the average value in the dendritic shaft (to compensate for ongoing fluorescence bleaching) and expressed relative to the baseline. Analysis was done blind with respect to the treatment the slices had received. (TIF) [file pbio.1001262.s007.tif]

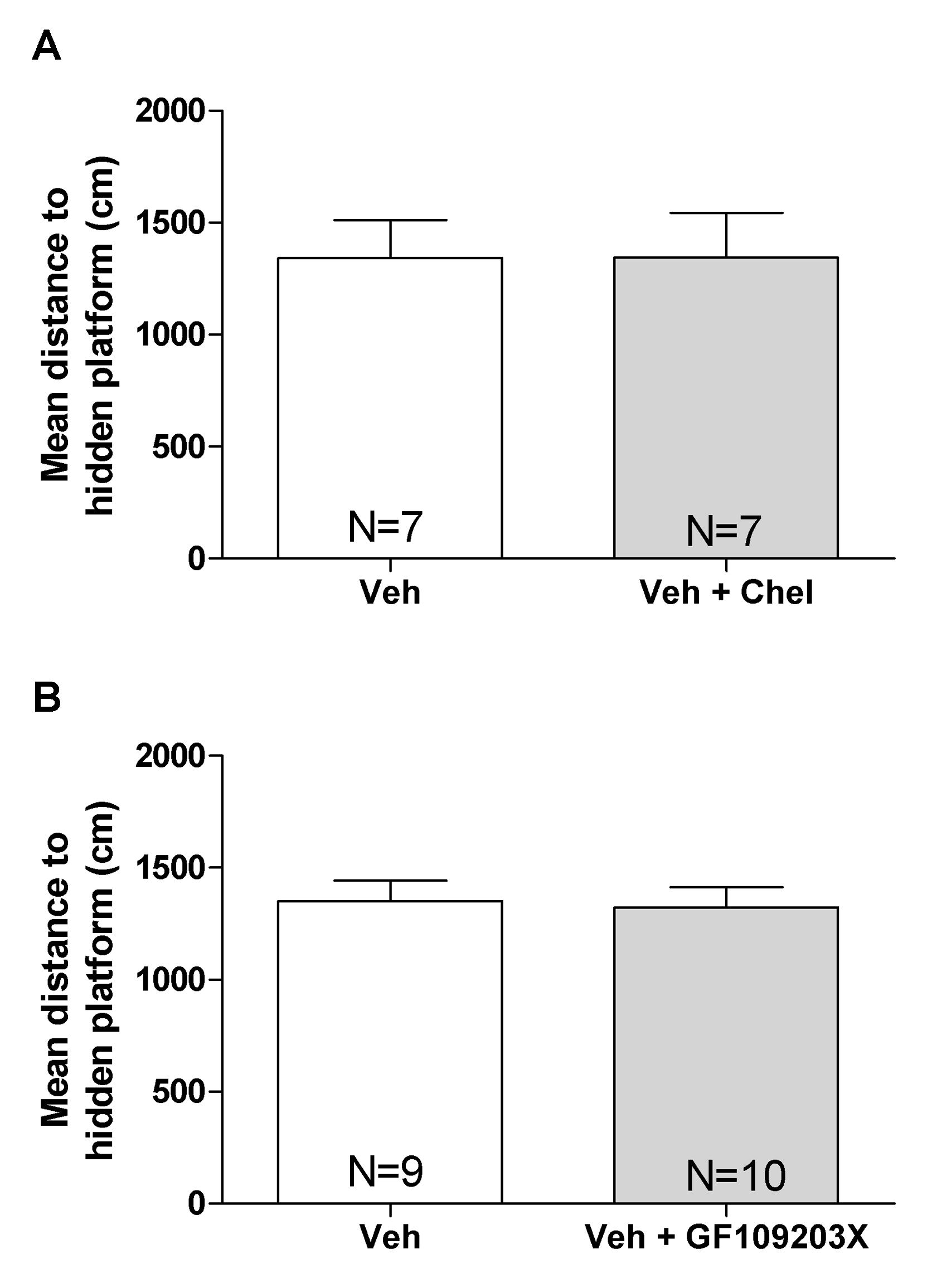

Supplement: Figure S8 — PKC inhibition does not alter spatial learning. Mean distances swam to find the hidden platform in the Morris water maze from vehicle- (white column) and PKC inhibitor-treated rats (gray columns; A, chelerythrine; B, GF109203X), over the eight training trials (four trials per day). N, the number of animals. (TIF) [file pbio.1001262.s008.tif]

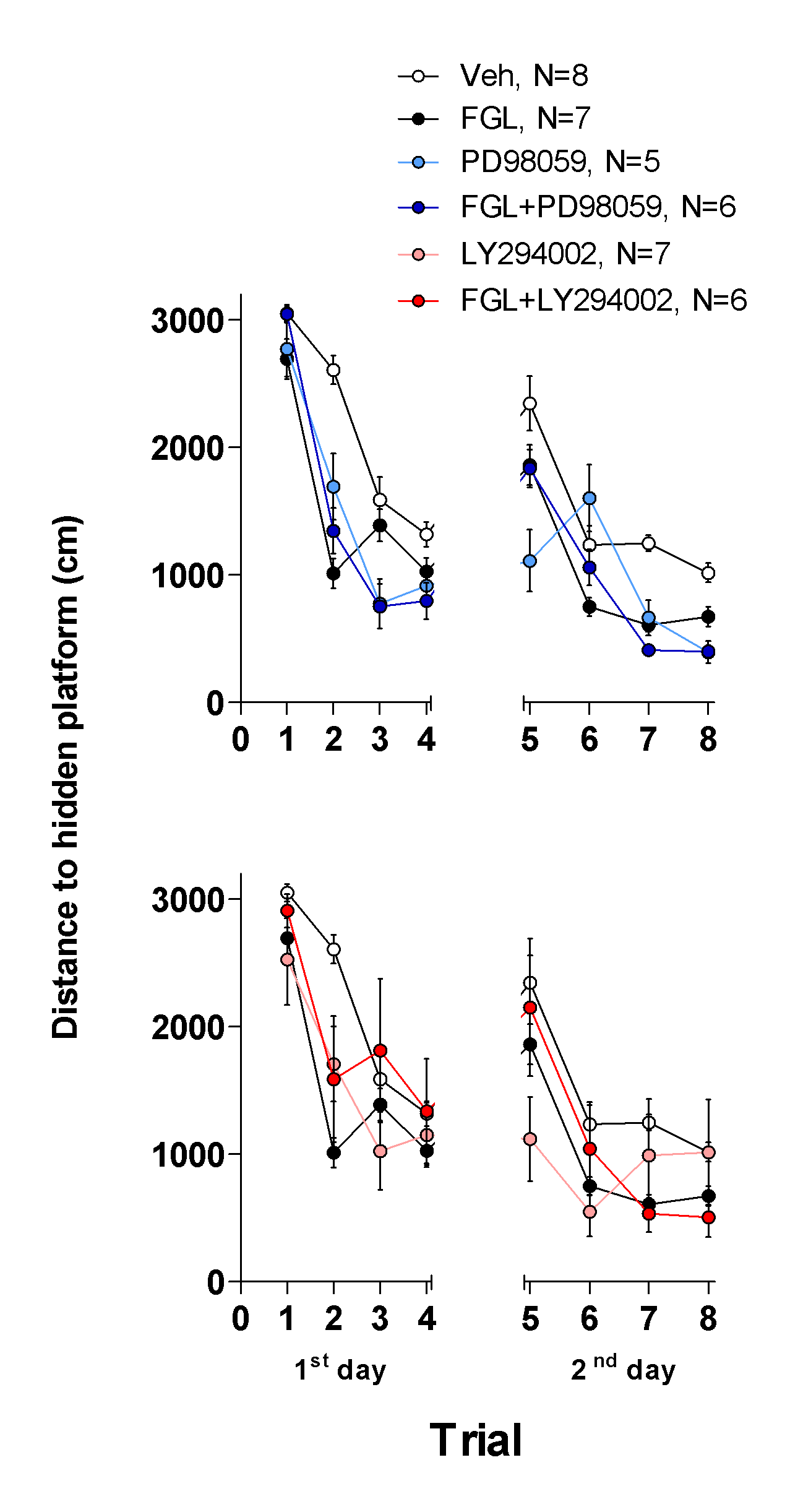

Supplement: Figure S9 — Spatial learning in the Morris water maze is altered by MEK and PI3K inhibitors. Mean distances traveled to find the hidden platform in the Morris water maze over the 2 training days (four trials each). N, number of animals. Rats injected with vehicle, FGL (20 µg), PD98059 (MEK inhibitor, 20 nmol), or LY294002 (PI3K inhibitor, 4.5 nmol), as indicated. (TIF) [file pbio.1001262.s009.tif]
