# Supplementary material for: Facilitation of AMPA Receptor Synaptic Delivery as a Molecular Mechanism for Cognitive Enhancement
Source: PLoS Biol. 2012 Feb 21;10(2):e1001262. doi: 10.1371/journal.pbio.1001262 (PMC3283560; doi:10.1371/journal.pbio.1001262)
Supplement: Text S1 — Additional experimental procedures are described in this supplementary text. (DOCX) [file pbio.1001262.s010.docx]

**SUPPORTING TEXT S1**

**Animals**

Three rats were housed per cage under controlled light (12 h/12 h light/dark cycle; lights on at 7 AM) and temperature (22 ± 2°C) conditions, and food and water were available *ad libitum*. A different group of rats was used for each experiment (behavior, biochemistry, morphology and electrophysiology), and all procedures that involved animals were performed in accordance with the guidelines of European Community Council Directives (86/609/EEC).

**Morris Water Maze**

*Spatial training.* Four different starting positions were situated at equal distances around the perimeter of the pool, and every rat used all four starting positions once on each day in a random sequence. A trial began by placing the rat in the water facing the wall of the pool at one of the starting positions. If the animal failed to find the hidden platform within 120 s, then it was gently guided to the platform by the experimenter. Once on the platform, the rat was allowed to remain there for 30 s and was then placed in a holding cage for 30 s until the beginning of the next trial. After the last trial, the rats were carefully dried with a towel and placed for 10 min in a waiting cage heated to 32 °C for 10 min. The rats were the returned to their home cages. The second training day was similar to the first. Statistical analyses were performed using SPSS version11 (Chicago, IL, USA), and the Morris water maze training data were analyzed across trials with one-way analysis of variance (ANOVA) using the training trial as the repeated measure. The distance swam to find the platform during each trial in the water maze was analyzed with a Bonferroni’s post-hoc test.

**Morphology**

*Intracellular injections of Lucifer Yellow.* Rats (n = 6/group) were anesthetized with pentobarbital (0.04 mg/kg) and transcardially perfused with 100 ml phosphate buffer (PB) followed by 300 ml of 4% paraformaldehyde (pH 7.4) prepared in the same buffer. The brains were removed from the skull, coded, and post-fixed in the same solution for 24 h. The codes were not broken until the quantitative analysis was completed. Coronal sections (150 µm) were cut on a vibratome and labeled with 4,6-diamidino-2-phenylindole (DAPI, Sigma D9542) for 5-10 min. Cells in the hippocampus (anteroposterior -3.8 to -4.3 mm from Bregma) were individually injected with 4% Lucifer Yellow (CH, Aldrich) in 1 M LiCl (pH 7.4) by passing a steady hyperpolarizing current through the electrode (-0.5 to -1.0 nA). The current was applied until the distal tips of each neuron fluoresced brightly. For each animal, half of the injected sections were processed for DAB (3,3'-diaminobenzidine) staining to count spines, and the other half were processed for fluorescence labeling to assess spine morphology.

*Tissue processing.* Following injection, the sections were processed with an antibody to Lucifer Yellow (1:400,000 in stock solution [2% bovine serum albumin, Sigma A3425; 1% Triton X-100, BDH 30 632; 5% sucrose in 0.1 mol/l phosphate buffer]) then with a biotinylated species-specific secondary antibody (Amersham RPN 1004; 1:200 in stock solution). For slices assigned to spine density analysis, the secondary antibody was visualized with a biotin-horseradish peroxidase complex (Amersham RPN1051; 1:200 in phosphate buffer), and DAB (Sigma D 8001) was used as the chromogen. Slices were mounted on glass slides in glycerol with azide, and the coverslip was then sealed with nail polish.

Slices used to determine spine morphology were first incubated in an anti-Lucifer Yellow antibody as described above and then with Alexa488 conjugated secondary antibody (Molecular probes, 1:1000) for 4 h. The sections were mounted on a glass slide in fresh ProLong Gold antifade reagent (Invitrogen, Eugene, OR). Slides were left for 24 h in the dark at room temperature for the mounting medium to cure, and the coverslip was then sealed with nail polish.

*Spine density*. Apical dendrites were traced with the Neurolucida 7.1 computerized data collection system coupled to an Olympus microscope (BX51), and using a 100× objective (Oil, NA 1.30, working distance 60 μm). The dendritic spine density of CA1 neurons was determined by tracing two oblique apical dendrites with all of their branches up to the distal tips and marking the spines while tracing. This was done for 80 neurons (5 neurons from each rat). All protrusions were considered spines and were included in the spine analysis, and no correction factors were applied to the spine counts. Dendrite tracing at high power allows all spines that protrude from the dendrites to be visualized (the DAB reaction product is transparent). The reconstructed data were exported to Neurolucida Explorer (MicroBrightField Inc., Williston, VT,) for quantitative analysis, and the spine density was automatically calculated by dividing the number of spines on a dendrite by the dendrite length.

*Spine morphology.* For confocal microscopy, one randomly selected apical dendrite was scanned from each CA1 pyramidal neuron (five neurons from each rat, eight rats from each group, 80 neurons in total). Imaging was performed on a Leica laser scanning multispectral confocal microscope (TCS SP5) using an argon laser. The image stacks (physical size 76.9 × 76.9 μm, logical size 1024 × 1024 pixels) consisted of 100-200 image planes, and a 63× glycerol-immersion lens (NA, 1.3, working distance, 280 μm, refraction index, 1.45) were used with a calculated optimal zoom factor of 3.2 and a z-step of 0.14 μm (voxel size, 75.1 × 75.1 × 136.4 nm). For each stack, the laser intensity and detector sensitivity were set so that the fluorescence signal from the spines occupied the full dynamic range of the detector. This meant that some pixels were saturated in the dendritic shaft, but no pixels were saturated within the spines.

*Image analysis.* After acquisition, the stacks were processed with a three-dimensional blind deconvolution algorithm (Autodeblur; Autoquant, Media Cybernetics) using 10 iterations to reduce the out-of-focus light. The three-dimensional image processing software IMARIS 5.0 (Bitplane AG, Zurich, Switzerland) was used to measure the spine head volume and neck length.

**Biochemistry**

*Western blot.* For determination of FGFR1 phosphorylation *in vitro*. Trex293 cells (Invitrogen, Eugene, OR) were stably transfected with human FGFR1 splice variant IIIc, with a *C*-terminal Strep II tag (IBA Biotech, Göttingen, Germany). Determination of FGFR phosphorylation after treatment of the cells with the FGL peptide or FGF1 (positive control) was then performed. Briefly, the Trex293 cells were stimulated with FGL at various concentrations. After the treatment of the cultures for 20 min, activated FGFR1-IIIc was immunopurified by incubating the lysates with agarose-coupled anti-phosphotyrosine antibodies (4G10-AC, Upstate Biotechnologies, Lake Placid, NY, USA) and analyzed by Western blot using antibodies against the StrepII tag (IBA Biotech). In each experiment, the same amount of protein was used for purification, and the experiments were repeated at least four times.

*ELISA.* For tissue preparation, the rats were decapitated 1 h after subcutaneous injection of FGL or vehicle, and the brains were kept on ice while the hippocampus was isolated. Tissue samples were coded and stored at -80ºC until use. The tissue was homogenized in 10 volumes of 0.32 M ice-cold sucrose and 5 mM HEPES buffer that contained a cocktail of protease inhibitors (Complete TM, Boehringer Mannheim, UK). The protein concentration of each sample was estimated by the Bradford method.

*Quantification of FGFR1 and TrkB phosphorylation.* Phosphorylation of FGFR1 and TrkB was quantified by ELISA. Briefly, the primary antisera were a polyclonal phosphospecific rabbit anti-FGFR-1 antiserum (pYpY653/654, diluted 1:100-Biosource International,) or a rabbit polyclonal anti-TrkB (pY515, diluted 1:100, Abcam). The secondary antibody was a peroxidase-conjugated anti-rabbit IgG (whole molecule conjugate; diluted 1:500; Sigma, Spain). All samples were assayed in triplicate, and the optical density was measured at a wavelength of 492 nm using a DigiScan Reader version 3.0 and DigiWIN software (ASYS Hitech GmbH, Austria). The average absorbance of three wells was subtracted from each reading.

**Electrophysiology**

The external solution (aCSF) contained 119 mM NaCl, 2.5 mM KCl, 1 mM NaH_2_PO_4_, 11 mM glucose, 26 mM NaHCO_3_, 4 M MgCl_2_, 4 mM CaCl_2_ and 2 μM 2-chloroadenosine, pH 7.4, and was gassed with 95% O_2_ and 5% CO_2_. Patch recording pipettes (4-7 MΩ) were filled with internal solution containing 115 mM CsMeSO_3_, 20 mM CsCl, 10 mM HEPES, 2.5 mM MgCl_2_, 4 mM Na_2_-ATP, 0.4 mM Na-GTP, 10 mM sodium phosphocreatine, 0.6 mM EGTA, and 0.1 mM spermine, pH 7.25. Bipolar stimulating electrodes were placed over Schaffer collateral fibers between 250 and 300 μm from the CA1 recorded cell, and synaptic responses were evoked with single voltage pulses (200 μs, up to 30 V).

**Pharmacological LTP induction**

This protocol was originally developed by the laboratory of John Lisman (Otmakhov et al., “Forskolin-induced LTP in the CA1 hippocampal region is NMDA receptor dependent”. J Neurophysiol, 91, 1955-1962, 2004). Briefly, slices are first equilibrated in aCSF. Then, they are perfused with the LTP induction cocktail, containing 0.1 μM rolipram, 50 μM forskolin and 100 μM picrotoxin, in aCSF lacking MgCl_2_ and 2-chloroadenosine. After 15 min, slices are perfused again with regular aCSF. This protocol has been shown to produce long-lasting, NMDAR-dependent synaptic potentiation of AMPAR-mediated responses (reference above).
